# Phenotypic disparity in Iberian short-horned grasshoppers (Acrididae): the role of ecology and phylogeny

**DOI:** 10.1186/s12862-017-0954-7

**Published:** 2017-05-04

**Authors:** Vicente García-Navas, Víctor Noguerales, Pedro J. Cordero, Joaquín Ortego

**Affiliations:** 1 Department of Integrative Ecology, Estación Biológica de Doñana (EBD-CSIC), Avenida Américo Vespucio 26, E-41092 Seville, Spain; 2 Grupo de Investigación de la Biodiversidad Genética y Cultural, Instituto de Investigación en Recursos Cinegéticos IREC (CSIC-UCLM-JCCM), Ronda de Toledo 12, E-13071 Ciudad Real, Spain

**Keywords:** Ecomorphology, Geometric morphometrics, Phenotypic evolution, Morphostatic radiation, Orthoptera, Tempo and mode

## Abstract

**Background:**

The combination of model-based comparative techniques, disparity analyses and ecomorphological correlations constitutes a powerful method to gain insight into the evolutionary mechanisms that shape morphological variation and speciation processes. In this study, we used a time-calibrated phylogeny of 70 Iberian species of short-horned grasshoppers (Acrididae) to test for patterns of morphological disparity in relation to their ecology and phylogenetic history. Specifically, we examined the role of substrate type and level of ecological specialization in driving different aspects of morphological evolution (locomotory traits, chemosensitive organs and cranial morphology) in this recent radiation.

**Results:**

We found a bimodal distribution of locomotory attributes corresponding to the two main substrate type guilds (plant vs. ground); plant-perching species tend to exhibit larger wings and thicker femora than those that remain on the ground. This suggests that life form (i.e., substrate type) is an important driving force in the evolution of morphological traits in short-horned grasshoppers, irrespective of ancestry. Substrate type and ecological specialization had no significant influence on head shape, a trait that showed a strong phylogenetic conservatism. Finally, we also found a marginal significant association between the length of antennae and the level of ecological specialization, suggesting that the development of sensory organs may be favored in specialist species.

**Conclusions:**

Our results provide evidence that even in taxonomic groups showing limited morphological and ecological disparity, natural selection seems to play a more important role than genetic drift in driving the speciation process. Overall, this study suggests that morphostatic radiations should not necessarily be considered as “non-adaptive” and that the speciation process can bind both adaptive divergence mechanisms and neutral speciation processes related with allopatric and/or reproductive isolation.

**Electronic supplementary material:**

The online version of this article (doi:10.1186/s12862-017-0954-7) contains supplementary material, which is available to authorized users.

## Background

Adaptive radiations, groups that have rapidly diversified from a common ancestor to exploit a wide suite of ecological niches, have intrigued evolutionary biologists for over a century [1, 2]. Considered as important biodiversity engines, these bursts of speciation are thought to arise from ecological opportunity in the form of vacant ecological niches that become available due to the colonization of new environments (spatial dispersal) or the acquisition of adaptive innovations that allow the access to novel niche dimensions (ecological dispersal) [3, 4]. Spatial and/or ecological dispersal can be driven by modifications of existing environments via climatic changes [4, 5]. When lineages first enter these new adaptive zones, morphological evolution should initially be rapid; as niche spaces become increasingly saturated, the rate of morphological evolution would be expected to slow down [6, 7]. A core prediction based on the above scenario (the so-called early-burst model) is the existence of early rapid diversification followed by a slowdown in net diversification over time [8–10]. Thereby, lineage and morphological diversification are frequently positively correlated [11, 12], but the opposite pattern (i.e., a negative relationship) has also been reported [13, 14]. Nevertheless, recent studies have shown that processes underlying phenotypic disparity and those generating species diversity can be uncoupled, suggesting that ecological opportunity is not the only diversification force [15–17]. For example, speciation by simple geographic isolation can generate a pattern of declining speciation trough time without the intervention of niche-filling processes [18]. On the other hand, speciation bursts can occur in multiple pulses linked to environmental changes (e.g., pulses of orogenic uplift or glacial retreat), thus eroding the diversity-dependent signature of diversification. This may explain why a large number of studies performed on radiating clades have failed to detect early-bursts of phenotypic evolution, despite some of them represent the most classic examples of adaptive radiation, including Darwin’s finches from the Galapagos Islands and *Anolis* lizards of the Caribbean Islands (reviewed in [6]).

Recently, it has been recognized that there are many different types of evolutionary radiations and not all adaptive radiations conform to the early-burst model [19, 20]. For example, under a repeated radiation scenario, it is expected that subclades tend to resemble each other in morphological disparity and, contrary to that predicted under the early-burst paradigm, morphological evolution should not necessarily be initially rapid. This kind of adaptive radiation (“iterative radiation”) may arise in systems dominated by constraints, such as when evolution within clades is driven by repeated adaptation to similar environments [21–23]. This model of radiation exemplifies that episodes of ecological opportunity can be recurring over the evolutionary history of a lineage [24]. Beyond the epithet “adaptive”, non-adaptive radiations (or morphostatic radiations) constitute another form of radiation that remains largely unstudied [25–27]. Non-adaptive radiations arise through processes that are unrelated to niche exploitation and the resulting species usually exhibit low morphological disparity and allopatric distributions [28]. For example, non-adaptive radiations driven by sexual selection result in new species that are ecologically similar to their ancestors such are the case of Hawaiian *Laupala* crickets [29]. The boundaries of adaptive and non-adaptive radiations are sometimes diffuse and it has been suggested that both processes are extremes along a continuum [30]. Thus, some lineages may exhibit features consistent with these two concepts [31, 32].

**Fig. 1:**
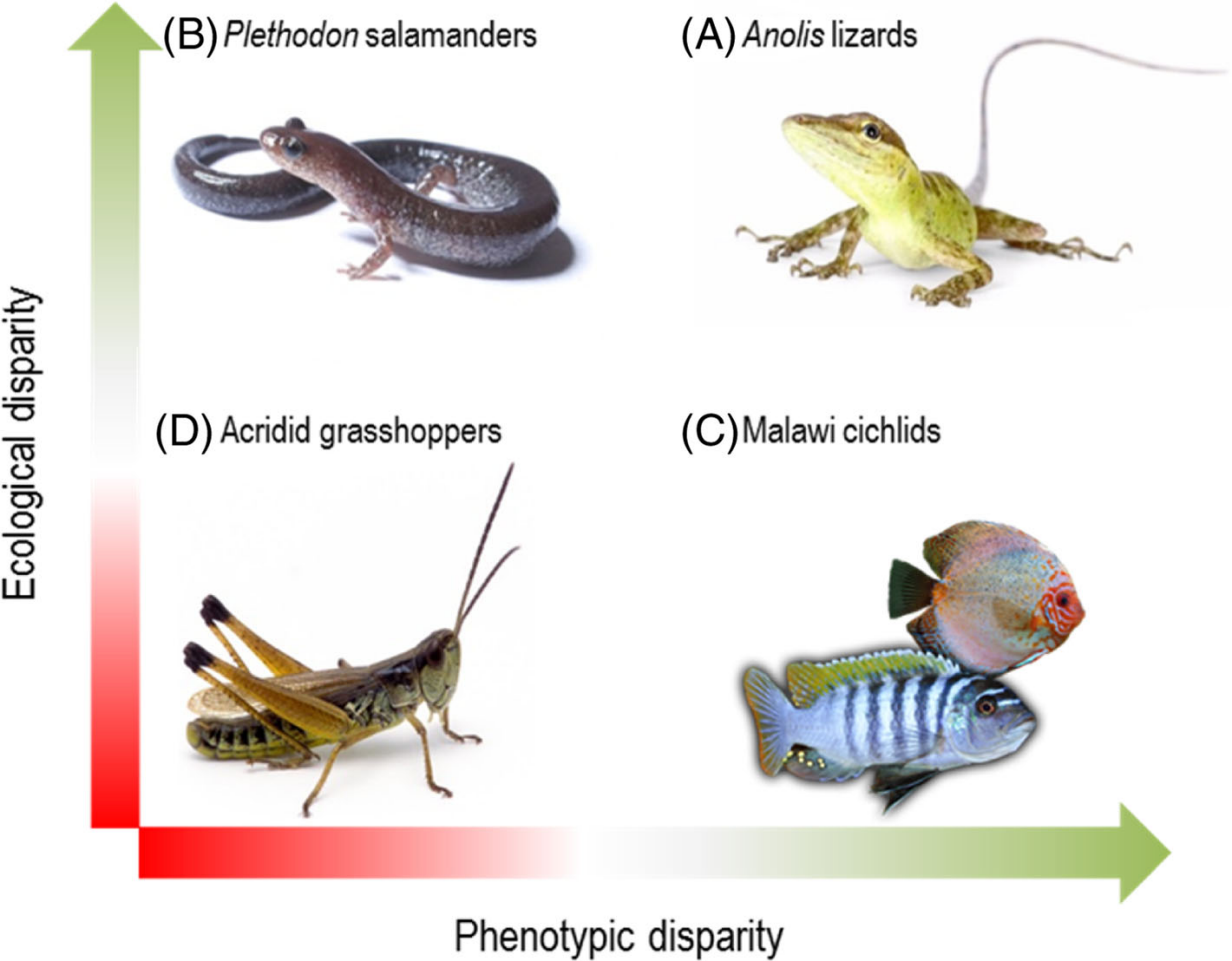
The axes of evolutionary radiation. Clades can show (**a**) strong phenotypic and ecological disparity like *Anolis* lizards or Hawaiian honeycreepers. Some clades (**b**) exhibit low morphological disparity despite of they have a wide variety of ecotypes (e.g., *Plethodon* salamanders) whereas others (**c**) present considerable phenotypic disparity, but little ecological disparity (e.g. African lake cichlids) suggesting a prominent role of sexual selection (disruptive or diversifying) as driving force in the speciation process. Clades (**d**) with limited morphological diversification and low ecological disparity (e.g., Acridid grasshoppers, *Bythinella* spring snails or Muroid rodents) constitute the most extreme cases along these axes and therefore, they have been frequently regarded as non-adaptive radiations

Striking radiations showing extraordinary phenotypic divergence, most of them comprising clades with restricted geographic distributions such as islands or ancient lakes, are over-represented in the literature on evolutionary radiations [33] (Fig. 1). Conversely, there is a paucity of studies analyzing the tempo and mode of trait evolution on species-rich clades that present little phenotypic disparity and that are more common among continental radiations. In this sense, recent radiations with limited morphological variability provide valuable case-studies for a comprehensive understanding of the evolutionary mechanisms that drive speciation (Fig. 1). However, despite of morphostatic radiations are likely not the exception but the rule within many groups such as invertebrates, this form of radiation remains largely unstudied. Short-horned grasshoppers (Acrididae), a superfamily that comprises over 6600 species [34], provide an excellent opportunity to study disparification dynamics in an evolutionary radiation exhibiting little apparent adaptive phenotypic disparity. According to a recent study, this group seems to have undergone a significant increase in diversification rate with little extinction, with the major diversification events occurring during the Cenozoic after the Cretaceous-Paleogene boundary [34]. Song and colleagues [34] suggested that the emergence of a new niche space (open grasslands) during the Cenozoic and the subsequent colonization of new habitats may have prompted an explosive adaptive radiation in short-horned grasshoppers. Later, during the Pleistocene, the most speciose acridid subfamily (Gomphocerinae) may have undergone one or several independent radiations due to the evolution of complex species-specific acoustic signals in different clades [35]. Contrary to that predicted under the adaptive radiation model, short-horned grasshopper species do not seem to exhibit remarkable morphological adaptations to different feeding habitats and environments. The existence of a low degree of morphological variation does not necessarily imply an absence of adaptation to different microhabitats. For example, plethodontid salamanders show limited morphological diversification in spite of presenting an extensive array of ecotypes ranging from arboreal to aquatic or fossorial species [36, 37]. Nevertheless, acridid grasshoppers seem to be rather conservative in microhabitat usage and, probably, the main differentiating factor among species is the substrate type wherein they perch; some species can be found on the plant canopy whereas others remain on the ground. Although most studies have focused on the relationship between microhabitat and morphology [38–40], it is likely that, at a finer scale, substrate type can also impose different selective pressures leading to the evolution of more or less subtle morphological and behavioral adaptations. The ability of species to exploit a range of habitats (niche breadth) could be also correlated to key morphological traits like forelimb length or cranial morphology as evidenced by studies on other taxa (see [41] and references therein).

In the present study, we used a time-calibrated phylogeny of 70 species of Iberian short-horned grasshoppers to test for patterns of morphological disparity in relation to their ecology and phylogenetic history. Specifically, we aimed at testing whether the major morphological changes occurred early in the acridid’s diversification history or if random-walk patterns (“morphological drift”) or other processes (e.g., evolutionary constraints) have driven the diversification of phenotypic attributes in this group. We also examined the potential role of substrate use and the level of ecological specialization (i.e., niche breadth of a given species) in shaping morphological attributes (locomotory traits, chemosensitive organs and cranial morphology) in this invertebrate radiation. If substrate type and ecological specialization imposes a selective pressure across taxa, it would result in the same solution -phenotypic optima- multiple times, leading to convergence among species even between distantly-related clades.

## Methods

### Taxon sampling

The Iberian Peninsula is renowned for its high level of biodiversity and number of endemic species, and also as one of the main refugial areas in Europe during the Pleistocene Ice Ages [42, 43]. Most of species included in this study are endemic to the Iberian Peninsula or have a distribution restricted to Iberia, France and North Africa. Our dataset included 70 taxa of short-horned grasshoppers belonging to four different subfamilies; slant-faced grasshoppers (subfamily Gomphocerinae, 43 spp.), band-winged grasshoppers (subfamily Oedipodinae, 17 spp.), spur-throated grasshoppers (Catantopinae, 5 spp.) and 5 spp. belonging to different subfamilies (Calliptaminae ×3 spp., Dericorythinae and Eyprepocnemidinae) (Additional file 1: Table S1). Our dataset accounts for around three quarters of all extant species of acridid grasshoppers that have been recorded in the Iberian Peninsula [44]. Iberian acridids do not descend from a single common ancestor and, thus, they do not constitute a local radiation in this region, but a paraphyletic group [34]. However, it should be noted that several recent studies have pointed out that analyses of adaptive radiation do not require to include all descendants of a particular common ancestor, as such requisite is overly restrictive (see e.g. [19]). In fact, no theory of adaptive radiation predicts monophyly [4].

### Phylogeny reconstruction

We sequenced four mitochondrial gene fragments: cytochrome c oxidase subunit 1 (COI), NADH dehydrogenase subunit 5 (ND5), 12S rRNA (12S) and a fragment containing parts of 16S rRNA (16S). For some taxa we failed to obtain reliable sequences, so we complemented our dataset with sequences obtained from previous studies [45, 46]. Sequences were aligned in MAFFT online version 7 [47] and concatenated using Sequencematrix 1.7.8. [48]. We calculated the best-fit models of nucleotide substitution for each of the four genes using jModelTest 0.1.1 [49]. The TIM2 + I + Γ substitution model was selected for 12S, GTR + I+ Γ for 16S, TrN + I+ Γ model for ND5 and TPM3uf + I+ Γ was selected for COI. More details about these procedures are given in [50].

Phylogenetic inference was carried out under both maximum likelihood (ML) and Bayesian frameworks. Maximum likelihood analyses were conducted with two search replicates and 1000 bootstrap replicates using GARLI version 2.0 [51]. Bayesian inference analyses were conducted in BEAST 1.8.0 [52] in order to estimate a time-calibrated phylogeny. We used an uncorrelated lognormal relaxed-clock model and applied a Yule process as tree prior. Two calibration points were used in order to calibrate the tree based on absolute times. We employed as a first calibration point the split between Gomphocerinae and Oedipodinae, estimated to have occurred ~100 Mya ago. This estimate is based on dated ancient cockroach fossils [53]. As a second calibration point, we used the divergence between *Sphingonotus azurescens* (mainland species) and *S*. *guanchus* (endemic to La Gomera Island, Canary Islands), whose divergence is estimated to have occurred around 3.5 Mya [54]. *Sphingonotus guanchus* was only included in BEAST analyses for calibration purposes. Note that dating should be treated with caution as our calibrations are based on previous estimates. We ran analyses for a total of 100 × 10^5^ generations, with a sampling frequency of 1000 generations. We ensured that replicated analyses converged (effective sample size values >200) using Tracer 1.4.1. Tree and log files (9500 trees after a 5% burn-in) of the two runs were combined with LogCombiner 1.4.7 [52], and the maximum clade credibility (MCC) tree was compiled with TreeAnnotator 1.4.7. The obtained tree topologies were rather similar and consistent with previous studies [46]. See [50] for more details.

### Morphometric analyses

#### i) Locomotory morphology

Our dataset included 316 preserved specimens representing 3 to 5 male adults for 70 species of short-horned grasshoppers. We gathered a dataset of five morphological attributes of known correlation with locomotor performance and ecology [55]. Morphological measurements included: (1) structural (i.e., head + thorax) body length, (2) tibia length, (3) femur width, (4) femur length, and (5) forewing (tegmina) length. All measurements were taken by the same observer (VGN) using a ZEISS stereomicroscope (SteREO Discovery V.8; Carl Zeiss Microscopy GmbH, Germany). Because disparity in body size and level of sexual size dimorphism has been addressed in a previous study [50] and the former trait (body size) is strongly correlated with other morphological variables, we size-corrected the remaining four variables using structural body length as size measurement. The relative length and relative width of the femur are inversely correlated (see Additional file 1: Figure S1), so we calculated a width/length femur ratio. As a result, our final dataset was composed of three variables related with locomotory morphology: tibia length, femur width/length ratio and tegmina length. Tegmina are modified leathery forewings present in some insects like grasshoppers. The major role of this structure is that of protecting the hindwings (and therefore, hind- and forewing length are highly correlated; *p* < 0.001, *R* ^2^ = 0.98). These also have an aerodynamic function and thus can be considered an informative trait about the dispersal potential of a species [56]. On the other hand, leg length is thought to determine the jumping performance of insects like grasshoppers, leafhoppers and froghoppers [57–59]. Thus, relative femur length can provide information on jumping ability in insects that use a catapult-like mechanism to jump. We performed a phylogenetic principal component analysis (*p*PCA) on these three variables using the R package *phytools* [60]. The phylogenetic principal component analysis yielded a single principal component (hereafter *p*PC*lm*) that explained 53.2% of the variance of our three morphological variables (loadings; tibia length: −0.518, width/length femur ratio: −0.737, tegmina length: −0.886). The positive extreme of *p*PC*lm* represents species with short wings and tibiae and thicker femora (e.g., *Pezotettix giornae*, *Podisma carpetana*), and the negative extreme of this axis represents species with large wings and tibiae and more stylized femora (e.g., *Chorthippus jucundus, Stethophyma grossum*). We also implemented a non-phylogenetic principal component analysis (PCA) as a recent study has suggested that using phylogenetic PC axes as trait data could bias results [61]. However, we found similar results by using both approaches (correlation pPCA vs. PCA; *p* = 0.028, *r* = 0.33).

#### ii) Antenna length

In conjunction with variables directly related to locomotor performance we also measured antenna length in a similar way as described above. Antenna length was corrected for body size, so we refer to relative antenna length hereafter. Members of the family Acrididae show relatively short and stout antennae in comparison with crickets and katydids. However, the length of antennae largely differs among taxa and these differences may be linked to the diet and degree of ecological specialization of each species [55]. Acridid grasshoppers recognize their host plant by sensorial stimuli, which are perceived through chemoreceptors located on the labrum and antennae [62]. Chemosensory sensilla on antennae express two classes of proteins involved in the recognition of odors and taste, and thus the antennae are thought to play an important role in host orientation and food selection [63]. In this vein, we would expect a greater development of this structure in taxa with a narrower ecological niche (i.e., specialist species) and species preferring to perch on plants, which may display higher sensitivity to plant odour perception [64].

#### iii) Head shape

**Fig. 2:**
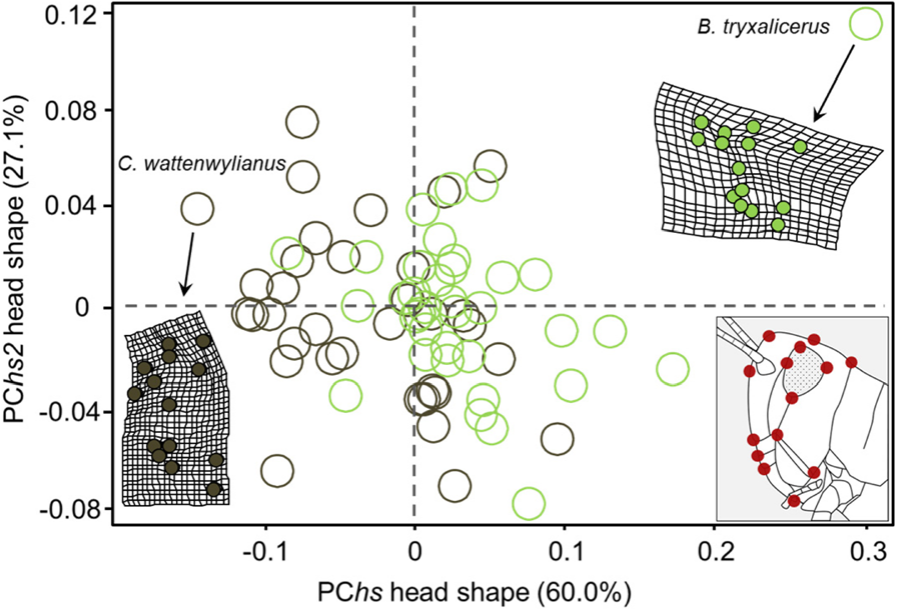
Scatterplot of the two first principal components of head shape variation (PC*hs* vs. PC*hs*2). Green and *brown circles* indicate plant-perching and ground-perching species, respectively. Thin-plate spline deformation grids for the two most extreme cases relatives to the overall reference shape (*C. wattenwylianus* and *B. tryxalicerus*) are shown. A picture illustrating the position of the 14 landmarks used to characterize head shape variation is also represented in the *right-bottom* corner of figure

Different selective pressures linked to a plethora of factors both intrinsic (e.g., genetic) and extrinsic (e.g., predation risk, habitat structure) can play a role in driving the evolution of head shape. The functional trade-offs that shape head morphology may be related to foraging, locomotory and anti-predator strategies, which may differ among species that move in different microhabitats, for example, between species that climb vertical structures and ground-dwelling taxa [65]. Here, we aimed at examining head shape variation in relation to ecological factors and phylogenetic history. To that end, we photographed a total of 221 specimens (3–4 individuals per species) using a ZEISS stereomicroscope and the ZEISS image analysis software (ZEN2). We established the scale and digitalized landmarks from each photograph using the function ‘digitize2D’ in the R package *geomorph* [66]. We selected 14 homologous landmarks which capture the outline of the head and the size and relative position of the eyes (see inset in Fig. 2). These landmarks were subjected to a generalized Procrustes analysis (GPA) wherein all specimens are translated to the origin, scaled to unit centroid size and optimally rotated until the coordinates of corresponding points align as closely as possible [67]. The resulting coordinates in the tangent space represent the head shape (side view) of each specimen. From these coordinates, we calculated an average set of landmark coordinates for each of the 70 species. We averaged each *x*- and *y*-coordinate, also calculating the species’ average centroid size. We then performed a PCA on the aligned Procrustes coordinates using the function ‘plotTangentSpace’ in *geomorph*. The first two PC (PC*hs* and PC*hs*2) accounted for 87.1% of shape variation in the sample (60.0 and 27.1%, respectively). We inspected how head shape was related to the log of centroid size (a size estimate) using the function ‘procD.allometry’. Head shape and centroid size did not show a linear relationship and thus, the allometric component of our data can be considered negligible.

### Ecological data

We aimed to examine the evolutionary relationship between grasshopper morphology (locomotory morphology, relative antenna length, and head shape) and substrate type. For this purpose, we categorized each species on the basis of substrate preference (ground vs. plant) from the literature (e.g. [68, 69]) and our own personal observations. In addition, we examined the association between our morphological variables and the level of ecological specialization (i.e., the niche breadth of a given species). Ecological specialization refers to the ability of a species to exploit a range of resources and its capacity to use each one as result of evolutionary trade-offs (the “jack-of-all-trades is master of none” hypothesis; [70, 71]). Here, we used the ‘Paired Difference Index’ (PDI) as an estimator of ecological specialization [72, 73]. PDI values were calculated from a species-habitat matrix in which we rated the level of association of each species (from complete generalist, 0, to complete specialist, 3) with the nine most common habitats in which the studied species can be found. The PDI is a robust specialization index which takes into account not only the number of resources used by a species, but also the strength of the association between the species and its resources [73]. Scores of species-habitat association were obtained directly from the literature and our own personal observations. PDI values were computed using the R package *bipartite* [74].

### Phylogenetic comparative analyses

We assessed the phylogenetic signal of our focal variables (locomotory morphology, relative antenna length, and head shape) in order to test if these traits tracked the evolutionary history of the group and were not randomly distributed across taxa. We computed Pagel’s lambda (λ; [75]) for the first two principal components of head shape (PC*hs* and PC*hs*2), and for the two remaining variables, locomotory morphology (*p*PC*lm*) and relative antenna length, in *phytools* [76]. Pagel’s λ can take a value from 0, which means that phylogeny has no impact on the distribution of the trait and the values can be treated as independent, to a value of 1, which means that phylogeny fully predicts the distribution of the trait. We then mapped the evolution of these continuous traits on our phylogeny by using the function ‘contMap’ in *phytools*, which estimates states at internal nodes using ML and interpolates the states along each edge following [77].

We examined the association between the two ecological factors (substrate type and degree of ecological specialization) and our morphological variables (relative antenna length, PC*hs* and *p*PC*lm*). First, in order to test if substrate type (“ground-perching” vs. “plant-perching”; categorical variable) has influence on these variables, we performed phylogenetic ANOVAs using the function ‘phy.anova’ (10,000 simulations) in the R package *geiger* [78]. Because substrate type largely varies among subfamilies (most gomphocerine species are plant-perching species whereas most of the remaining species stay on the ground) we performed a second phylogenetic ANOVA only including Gomphocerinae species (*n* = 43). Thereby, we assessed the influence of substrate type on morphology while controlling for the non-independence of the data because of shared ancestry. Secondly, we analyzed the relationship between our morphological variables and the level of ecological specialization (PDI index; continuous variable) using phylogenetic generalized least squares (PGLS_λ_). PGLS regression analyses were performed using the *caper* package [79] and graphically visualized by means of phylogenetically independent contrasts (PICs) computed using the PDAP:PDTREE module in Mesquite v.3.04 [80].

### Disparity analyses

In order to visualize the relationship between phylogeny and taxon distribution in the morphological space, we built a ‘phylomorphospace’, which projects the branches of a phylogenetic tree into a 2D morphospace defining trait variation among species [81]. When subclades occupy limited morphospace it means that most disparity is accounted for by early divergence (early-burst of trait evolution), while if subclades occupy large regions of the morphospace (i.e., stronger overlap) it means that morphological diversity (‘disparity’) is largely explained by recent divergence. We generated a phylomorphospace by plotting head shape against locomotion morphology (PC*hs* vs. *p*PC*lm*) using *phytools* [60]. We also generated a second morphospace from the two first principal components for head shape (PC*hs* vs. PC*hs*2).

We investigated how morphological disparity has accumulated over the group’ evolutionary history. We evaluated the rate of morphological evolution in relation to lineage diversification by means of disparity-through-time (DTT) analyses [82], as implemented in the *geiger* package [78]. We compared observed relative disparity with average phenotypic disparity simulated under Brownian Motion (1000 simulations) in order to test whether disparity was accumulated during the early or recent history of the group. When the rate of morphological evolution is constant, as expected under BM, the DTT curve is expected to decline linearly toward zero through time. Under an early-burst model, disparity will decline sharply and much earlier, while if evolution within subclades is fast the observed DTT curve will fall above the Brownian profile. In order to quantify the magnitude of the difference between the null disparity profile computed under BM, and the observed disparity profile from our data, we computed the so-called morphological disparity index (MDI) statistic using the *geiger* package [78]. A positive MDI value is indicative of greater than expected subclade disparity (i.e., disparity distributed primarily within subclades) whereas negative MDI values indicate greater than expected subclade disparity (i.e., disparity distributed primarily among subclades). A negative MDI is characteristic of adaptive radiations, as rapidly diversifying taxa are expected to evolve distinct morphologies in response to new adaptive zones and slow once niches are filled. DTT analyses were performed twice; by including all taxa and only including species of the subfamily Gomphocerinae. We then ran a node height test [83], which tests for accelerations or decelerations in trait evolution, by comparing the independent contrasts for a trait with the respective node height (i.e., relative age). A significant negative relationship between absolute contrast value and node age supports the hypothesis of adaptive radiations as it would imply that species are dividing niche space more finely through time, consistent with a niche-filling model. Node height tests were performed using the R package *caper* [79].

### Tempo and mode of morphological evolution

Evolutionary models are conceived to infer the different possible processes shaping phenotypic evolution and provide a method of testing different predictions regarding the tempo and mode of evolution. We assessed the fit of four evolutionary models to our phenotypic variables using the functions ‘fitContinuous’ and ‘OUwie’ implemented in the R packages *geiger* [78] and *OUwie* [84], respectively. We fitted three different single-rate models; Brownian-Motion (i.e., diffusive drift), Early-Burst (i.e., exponential declining of evolutionary rates) and single-optimum Ornstein-Uhlenbeck (i.e., bounded evolution around a single phenotypic optimum), and a multi-peak Ornstein-Uhlenbeck model. Brownian-motion (BM) operates under the assumption that trait evolution proceeds a random walk wherein trait variance across lineages accumulates proportional to time (single-rate model; [77]). The Early-Burst (EB) model predicts rapid morphological disparity early in the radiation, followed by a slowdown in the diversification rate as ecological niches are filled over time [82]. Support for the single-optimum OU model (OU1) would imply that there is a single phenotypic optima (θ) for all taxa. This model is often associated with a process of stabilizing selection in which variation of phenotypic traits revolves around one or more stationary peaks [85]. Lastly, we assessed the fit of a multivariate OU model (OUVM) with separate morphological optima and separate random walk variances (σ^2^) for each substrate guild (1: plant, 2: ground), and one global parameter (α), which determines the strength of selection towards those optima. For the multi-peak OU model we determined the possible ancestral substrate regimes along the internal branches of the phylogenetic tree using the Stochastic Mutational Mapping on Phylogenies (SIMMAP) tool in *phytools*, and sampled 500 character histories in order to incorporate evolutionary uncertainty. We compared the fit of the BM, EB, OU1 and OUMV models using the Akaike information criterion corrected for small sample size (AICc), which can be employed to compare models that differ in the number of parameters and therefore have non-comparable likelihoods.

## Results

### Phylogenetic signal and ancestral state reconstruction

All morphological variables except PC*hs*2 showed a significant phylogenetic signal suggesting conservative evolution for these traits (relative antenna length, λ = 0.837, *p* < 0.001; *p*PC*lm*, λ = 0.575, *p* = 0.003; PC*hs*, λ = 0.883, *p* < 0.001; PC*hs*2, λ ~ 0, *p* ~ 1). Visual simulation of trait evolution using ancestral character reconstruction confirmed this pattern; species in the same subfamily tend to share morphological traits (see e.g. Additional file 1: Figures S2-S3). However, within Oedipodinae, there is a greater morphological variability than in the Gomphocerinae group.

### Ecology-morphology association

**Fig. 3:**
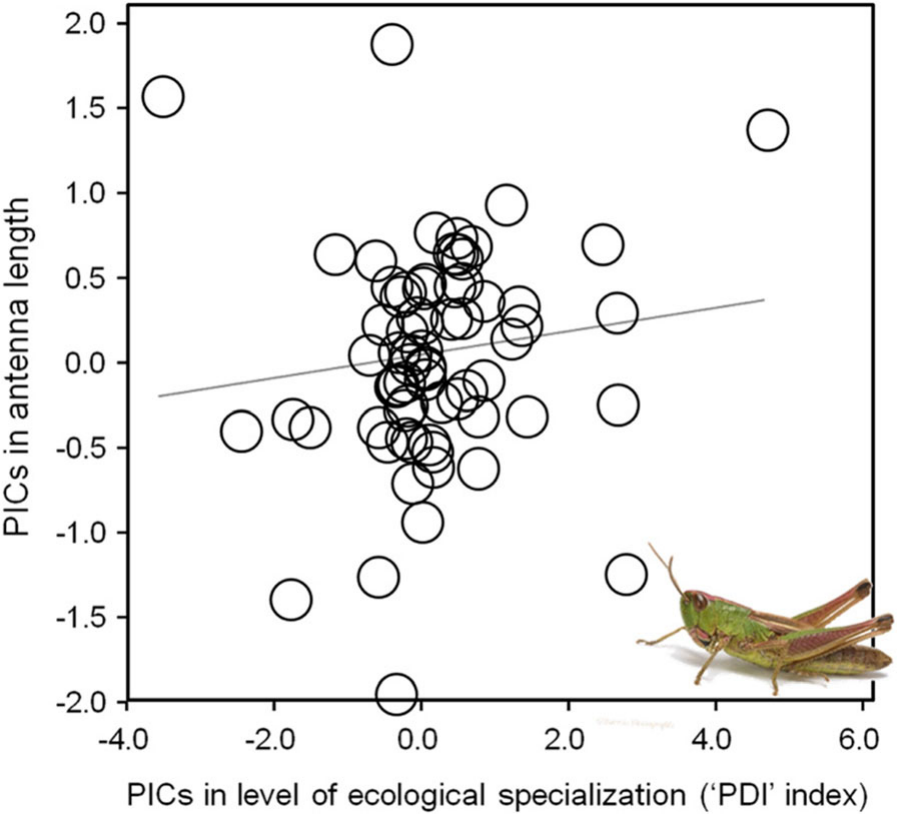
Relationship between relative antenna length and level of ecological specialization (‘PDI’ index) in short-horned grasshoppers represented in the form of standardized phylogenetic independent contrasts (PICs)

We found a marginally significant correlation between the relative length of antennae and the level of ecological specialization (PGLS; estimate: 0.304 ± 0.157, *t* = 1.92, *p* = 0.057); specialist species tend to have larger antennae than generalist species (Fig. 3). There was no significant association between either head shape (PC*hs*) or locomotory morphology (*p*PC*lm*) and the level of ecological specialization (*t* = 1.54, *p* = 0.128, and *t* = −1.37, *p* = 0.174, respectively).

**Fig. 4:**
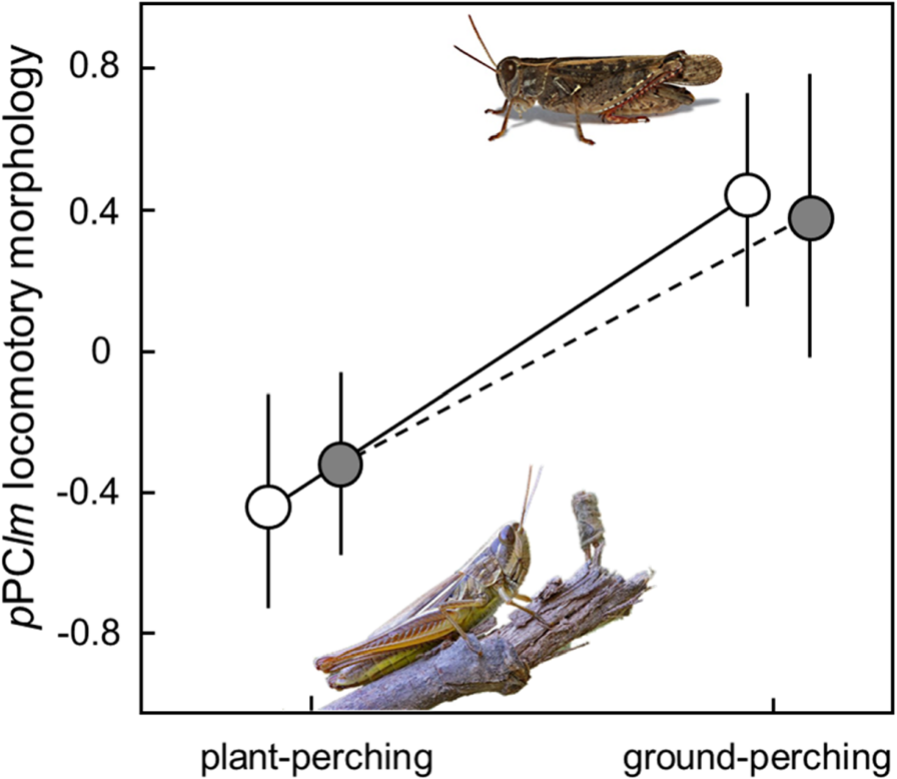
Differences (mean ± SD) in locomotory morphology between plant-perching and ground-perching species when considering all taxa (empty dots; *n* = 70 spp.) and only including Gomphocerinae species (filled dots; *n* = 48 spp.)

We found a significant effect of substrate type on head shape (PC*hs*) (phylogenetic ANOVA, *F*_1,68_ = 20.81, phylo-*p* = 0.029) indicating that plant-perching species exhibit a more conical head in comparison with ground species. However, when restricting our analyses to the Gomphocerinae subset, we did not find significant differences in head shape between both groups (*F*_1,42_ = 3.81, phylo-*p* = 0.25). This suggests that differences in head morphology between substrate types are mainly due to most of Oedipodinae being “ground-dwelling” taxa whereas most Gomphocerinae species are adept climbers that prefer to perch on plants instead of remaining on the ground. Regarding locomotory morphology (*p*PC*lm*), we detected marginally significant differences between plant-perching and ground-dwelling species (*F*_1,68_ = 14.60, phylo-*p* = 0.077); the former tend to exhibit larger tibiae and larger and thinner femora than ground-dwelling species, which possess a more stout morphology (Fig. 4). This difference remained marginally significant even when restricting our analyses to the Gomphocerinae subfamily (*F*_1,42_ = 9.00, phylo-*p* = 0.074); gomphocerine species that prefer to stay on the ground exhibit a slightly different locomotory morphology (short wings, thicker legs) in comparison with gomphocerine perching on plants. There were no significant differences in relative antenna length between ground and plant-perching species for either the entire dataset (*F*_1,68_ = 2.93, phylo-*p* = 0.31) or the Gomphocerinae subset (*F*_1,42_ = 0.33, phylo-*p* = 0.25).

### Disparity analyses

**Fig. 5:**
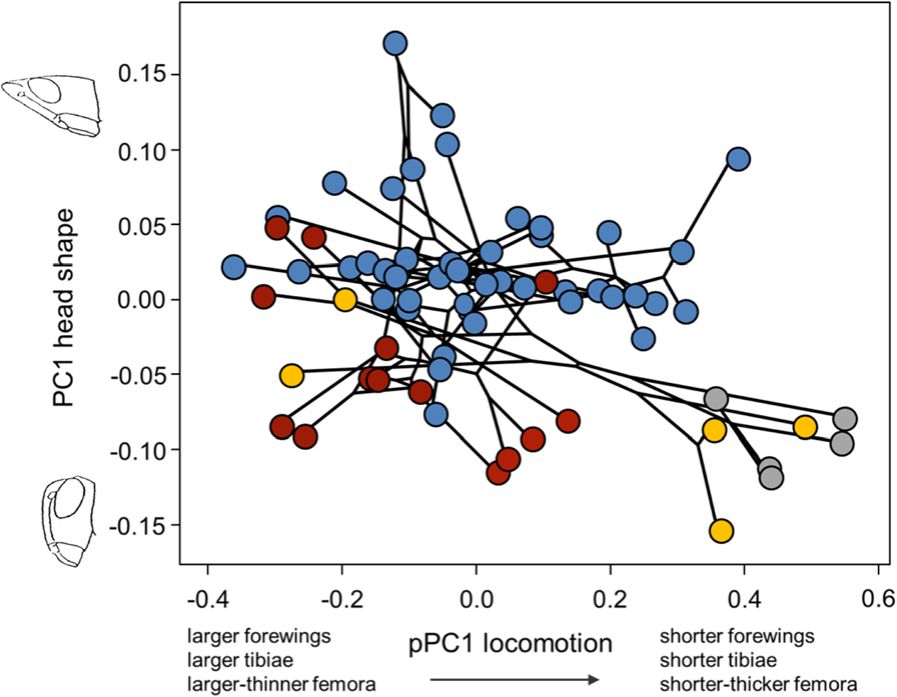
Phylomorphospace plot of head shape variation (PC*hs*) against locomotory morphology variation (*p*PC*lm*). *Dots* show mean values for each acridid species and colors indicate clade membership (*blue*: Gomphocerinae; *red*: Oedipodinae; *yellow*: Calliptaminae-Dericorythinae-Eyprepocnemidinae; *grey*: Catantopinae). *Black lines* show phylogenetic relationships among species. For illustrative purposes, *B. tryxalicerus* (the most extreme case for both variables) was not represented in the phylomorphospace but a scatterplot of PC*hs* vs. *p*PC*lm* including this species is provided in Additional file 1

Phylomorphospace plots of PC*hs* versus *p*PC*lm* and of PC*hs* versus PC*hs*2 indicate a moderate phylogenetic structuring of taxon distribution in the phenotypic space (see Additional file 1: Figure S4). We projected our phylogeny onto a plot defined by the first axis of head shape (PC*hs*) and the PC axis representing variation in locomotory morphology (*p*PC*lm*). We found that Catantopinae and Calliptaminae occupy a unique region of morphospace at the positive extreme of *p*PC*lm*, characterized by relatively small and stout limbs, whereas Gomphocerinae and Oedipodinae species showed a broader distribution along the morphospace (Fig. 5). Within the 2-dimensional space defined by PC*hs* versus PC*hs*2, we observed that the first axis, which represents variation between an elongated (slant-faced) to a straight head shape (60% of variation), separates most of gomphocerine species from the rest of taxa (Fig. 2). When discerning between both substrate types, we observed that plant-perching species (as most Gomphocerinae are) are more abundant in the right-side of the morphospace while ground species (23% of Oedipodinae falls within this category) tend to occupy the left-side (Fig. 4).

**Fig. 6:**
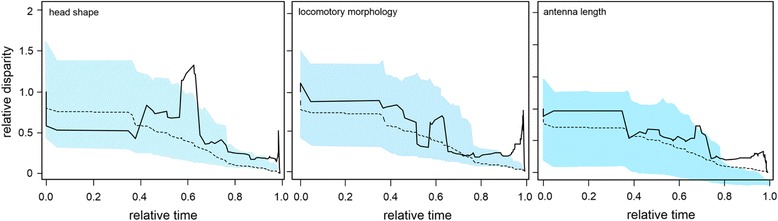
Disparity-trough-time plots for head shape (*left*), locomotory morphology (*middle*) and relative antenna length (*right*). Disparity profiles are indicated by *solid black lines*, average Brownian-Motion simulation by *dashed black lines*, and 95% confidence intervals from 10,000 BM simulations by *light blue* polygons

The DTT analyses yielded positive MDI statistics for all morphological variables (relative antenna length: 0.166, head shape PC*hs*: 0.070, locomotory morphology *p*PC*lm*: 0.137; Fig. 6), however the differences between both profiles were not statistically significant in either case (all *p*-values >0.05). It means that trait evolution did not significantly deviate from a BM model, which prevents us to state that disparity is concentrated within subclades (i.e., that closely related species differ considerably in morphology). For head shape, we found a higher pulse of trait disparification during the Paleogene, which is in agreement with previous studies in which it has been suggested that major lineages of Acrididae went through a major radiation in the Cenozoic [34]. In all cases we observed a departure of the observed DTT curves from BM expectations beyond the 95% confidence interval during the Pleistocene suggesting rapid acceleration in morphological diversification during this period. When restricting our analyses to this period (i.e., by including only the Gomphocerinae subfamily) we obtained again positive MDI values in all cases (relative antenna length: 0.355, head shape PC*hs*: 0.841, locomotory morphology *p*PC*lm*: 0.456), which implies that due to niche evolution subclades overlap (i.e., disparity is within subclades), and all contain a significant proportion of variation found through the Gomphocerinae group at a given time. The node-height test resulted in a positive but non-significant relationship between the absolute values of standardized length contrasts and node age for all morphological traits (PC*hs*: b = 0.011 ± 0.008, *t* = 1.36, *p* = 0.18; relative antenna length: b = 0.013 ± 0.007, *t* = 1.72, *p* = 0.09; *p*PC*lm*: b = 0.018 ± 0.01, *t* = 1.86, *p* = 0.07).

### Tempo and mode of morphological evolution

Results from the multivariate model fitting analysis support stabilizing selection (OU1, OUVM) as the most plausible evolutionary scenario for our morphological data (summarized in Table 1). According to the obtained AICc values, the OUMV model provides the best fit to head shape evolution (PC*hs*) followed by the BM model and lastly the OU1 and the EB models (Table 1). This means that head shape diversification may be driven by adaptive constraints, although a constant rate model could not be discarded. Indeed, when models of head shape evolution were tested exclusively on the Gomphocerinae, BM provided the best fit for the observed pattern (Table 1). Divergence of locomotion attributes was also best described by an OUMV model (Table 1), which suggests that acridid body morphology is tied to substrate type. This result was confirmed when restricting our analyses to the Gomphocerinae dataset, which allowed us to distinguish ancestral from derived similarity (Table 1). However, a single-peak OU model could not be excluded in this latter analysis. Regarding relative antenna length, the evolution of this trait was better described by a single-peak OU model either considering all species or restricting the analyses to Gomphocerinae (Table 1).

**Table 1:** Comparison of four evolutionary model fits, random-walk variances (σ^2^) and primary trait optima (in parentheses) for the three morphological variables: head shape (PC*hs*), locomotory morphology (*p*PC*lm*) and relative antenna length

	Model	AIC_c_	ΔAIC_c_	σ^2^_plant_ (θ_plant_)	σ^2^_ground_ (θ_ground_)
(a) All taxa					
PC*hs*	BM	−185.695	0.99	0.105	0.105
	EB	−183.508	3.36	0.105	0.105
	OU1	−183.927	2.94	0.129	0.129
	**OUVM**	**−186.869**	**0.00**	**0.042 (0.120)**	**0.198 (−0.013)**
*p*PC*lm*	BM	3.246	6.31	1.574	1.574
	EB	5.431	8.50	1.574	1.574
	OU1	1.734	4.80	3.117	3.117
	**OUVM**	**−3.066**	**0.00**	**−0.239 (−0.093)**	**0.060 (0.047)**
Antenna length	BM	−91.674	4.15	0.405	0.405
	EB	−89.489	6.33	0.405	0.405
	**OU1**	**−95.824**	**0.00**	**0.988**	**0.988**
	OUVM	−92.889	2.93	0.928 (0.7012)	0.928 (0.701)
(b) Gomphocerinae subset					
PC*hs*	**BM**	**−126.699**	**0.00**	**0.141**	**0.141**
	EB	−124.991	1.71	0.140	0.140
	OU1	−125.466	1.23	0.120	0.120
	OUVM	−120.905	5.79	0.142 (0.040)	0.112 (0.107)
*p*PC*lm*	BM	−5.034	5.57	2.234	2.234
	EB	−2.712	7.89	2.234	2.234
	OU1	−10.510	0.10	1.162	1.162
	**OUVM**	**−10.607**	**0.00**	**0.663 (−0.335)**	**1.073 (0.030)**
Antenna length	BM	−66.264	14.25	0.555	0.555
	EB	−63.949	16.56	0.555	0.555
	**OU1**	**−80.510**	**0.00**	**1.190**	**1.190**
	OUVM	−69.568	10.94	0.282 (0.841)	0.282 (0.744)

ΔAICc is the model’s mean AICc minus the minimum AICc between models. Bolded rows represent the best fit model as indicated by the lowest AICc score. Estimated phenotypic optima (θ) for ground and plant-perching regimes are shown where applicable. Analyses were performed for (a) all species and (b) only including the Gomphocerinae subset

## Discussion

Like some examples of non-adaptive radiations (*Plethodon*: [36], *Rattus*: [32]), acridid grasshoppers tend to retain a rather conserved body plan with little overt ecomorphological specialization among taxa (Fig. 6). However, we observed a greater morphological disparity within Oedipodinae in comparison with that observed for the Gomphocerinae, suggesting that the level of morphological resemblance among species differ between the two subfamilies. Gomphocerinae (slant-faced grasshoppers) constitute a very recent radiation, which is suspected to be mostly the result of divergent evolution of isolated populations induced by climatic oscillations during the Pleistocene [42]. Some gomphocerine species spread out from southern refugia and expanded their ranges northwards during interglacials, whereas many others (montane species) became restricted to high altitudes (“sky-islands”) due to their limited dispersal capacity [35, 86]. Hence, many Iberian species probably arose in association with these mountain ranges (Sistema Central, Betic ranges, Picos de Europa; [87]). At a smaller scale, the evolution of elaborate acoustic signals as premating reproductive isolation mechanisms (via sexual selection) may have allowed gomphocerine grasshoppers to diversify in regions in which ecologically similar and phylogenetically related species coexist [46, 86, 87]. Thus, it is likely that both divergence in allopatry [42, 86] and prezygotic isolation mechanisms [46, 88, 89] have played a crucial role in promoting speciation in this subfamily. However, our results should be interpreted cautiously because the present study assesses the dynamic of morphological evolution among a small fraction of the Acrididae’ overall diversity. Thus, our findings may reflect local processes (i.e. factors that have affected which species can occur together in the same regional theatre) rather than global mechanisms that operate across the entire clade.

### Patterns of morphological disparity through time

Our results do not support the existence of an early-burst of phenotypic diversification, adding to the number of study-cases showing no support for this evolutionary model [6, 7, 90, 91]. Obtained MDI values indicate that substantial amount of variance is clustered within subclades, pointing out to the existence of low phylogenetic niche conservatism. That is, there was no tendency of closely related species to be more similar to each other in ecomorphological traits than they are to more distant relatives as expected if some processes had constrained niche divergence among phylogenetically close species [92, 93]. This is in contrast to that predicted for taxa that experience early-bursts of diversification, where the partitioning of morphological disparity through time (disparification sensu [94]) is expected to be low [10, 82, 95]. Overall, DTT plots showed a general tendency for relative disparity in phenotypic attributes to decrease over time, but with two main pulses of increases in disparification during the Cenozoic and the Pleistocene. The observed pattern is not consistent with that predicted under an iterative radiation scenario wherein it should be expected the appearance of repeated diversification peaks [23]. The fact that acridids partitioned morphological disparity within rather than among clades suggests that acridid lineages did not evolve along distinct morphological trajectories through time. Thus, it is unlikely that acridid lineages explored different adaptive zones, which makes sense taking into account the limited morphological variability they exhibit.

### Evolutionary models of morphological divergence

Overall, results from the multivariate model fitting analysis summarized in Table 1 favored an Ornstein-Uhlenbeck model, indicating bounded evolution around a single (OU1) or two phenotypic optima (OUVM). Specifically, in relation to head shape, we found that a single model does not uniquely explain the evolution of this trait for the entire dataset; BM and OUMV were similarly informative. However, when models of evolution were tested exclusively on the Gomphocerinae subset, BM was the most informative model. It suggests that head shape evolution displays an idiosyncratic component and that, irrespective of substrate on which they rest, Gomphocerinae species have a more conical head shape in comparison with band-winged grasshoppers due to their phylogenetic legacy. On the other hand, our results indicate that locomotory morphology in Acrididae is inconsistent with a BM process, and has not evolved under a constant rate over time. A multivariate-peak OU (OUMV) model provided the best fit in both cases (when considering all taxa and after including only Gomphocerinae), which means that the evolution of this trait revolves around adaptive peaks.

In this context, it is possible that biomechanical constraints, which underlie much of locomotory morphology variation, impose restrictions on morphological diversification [96–98]. Specifically, our results suggest that stabilizing selection pulls the locomotory morphology towards two convergent adaptive optima (θ_plant_, θ_ground_) during the group’s evolutionary history. A key difference between the two substrate type regimes in terms of locomotory attributes relies on the different degree of development of femora (which has influence on jumping performance) and wings (which determines flight capability) that each guild of species exhibit (see more below). Interestingly, we observed a negative relationship between relative forewing length and relative femur width/length ratio in Gomphocerinae (see Additional file 1: Figure S5) pointing out to the existence of a trade-off between flying- and jumping-based dispersal investments in this group. With regard to antenna length, a single-peak OU model was the model that provided the best fit to the observed data, suggesting that antennae evolution has remained constrained by directional selection.

### Ecology-morphology association

We investigated the role of substrate type and level of ecological specialization in driving morphological evolution in our study group. The variation in structural characteristics within the habitat (substrate type, size and incline) has direct effects on animal locomotion and can lead to the evolution of morphological and behavioral adaptations (e.g., anti-predator strategies) [99]. For example, stick insects (order Phasmatodea) of New Caledonia and New Guinea (“land lobsters”) are ground-dwelling species and show a distinct ecomorph; they are flightless and exhibit a stocky body form and thick hind legs. This is in contrast with the majority of stick insects, which are solitary canopy-dwellers and show an instantly recognizable morphology (stick-like bodies with large and thin legs) [100]. In particular, substrate type may be especially important for small animals that jump using a catapult-like system such as grasshoppers and leafhoppers [101]. Here, we found that plant-perching grasshoppers tend to exhibit a slender morphology in comparison with those species that prefer to stay on the ground, which show a more compressed body (shorter and thicker femora). This result is consistent with that reported by [58] in a study with leafhoppers. These authors suggested that insect species that jump from plants have longer legs because it reduces the amount of energy lost to bending the leaf, whereas species that jump from a stiffer substrate can have shorter-legs [58]. Hence, in species living on plants, natural selection would have favored long hind legs because these allow short preparation times when an emergency jump is necessary [102]. The existence of moderate differences in morphology between ground-dwelling and plant-perching species was confirmed when restricting our analyses to the Gomphocerinae subset. Despite of members of the subfamily Gomphocerinae exhibit low variability in morphology (and as a result of this phenotypic conservatism some species can be almost only distinguished by song attributes), gomphocerine species perching on plants tend to present a less rotund morphology -larger and thinner legs- in comparison with gomphocerine species that prefer to stay on the ground, which show squat bodies and short limbs. Our results suggest that there are two possible ways to achieve superior jumping capabilities: to evolve longer limbs or to evolve more muscular limbs (as stronger legs will produce more acceleration) [102]. This finding is in agreement with that reported by [96] in a study with anole species, which also seem to face trade-offs that prevent them from simultaneously optimizing different skills of jumping ability (larger vs. thicker legs).

Head shape differed significantly between ground- and plant-species across taxa. Plant-perching species show a sharper head than ground-species, which may be favored by natural selection as this head shape facilitates camouflage against potential predators by making it difficult to see the mimic against the surroundings (i.e., background matching) [103]. However, this difference between both categories become non-significant within the Gomphocerinae, which implies that differences in head shape between ground- and plant-species have to do with its different phylogenetic history per se rather than with the possible existence of a substrate type effect. In band-winged grasshoppers (subfamily Oedipodinae), the orientation of the face is usually nearly vertical (straight head shape) whereas species of the subfamily Gomphocerinae (also known as slant-faced grasshoppers) show a sharper profile, that is, a more conical head.

Lastly, we also found a marginally significant association between the relative length of antennae and the level of ecological specialization. Larger antennae can bear a higher number of chemoreceptors, which would be favorable for grasshoppers in the search for shelter or food [104]. Hence, in those species that can thrive only in a limited range of habitats or have a limited diet (specialist species), the development of sensory organs may be favored, whereas in species with a broader ecological niche breadth (generalist species) selection for larger antennae may be weaker. Thus, the length of antennae in short-horned grasshoppers could be employed as a proxy for the degree of ecological specialization in terms of habitat requirements of a given species.

## Conclusions

Short-horned grasshoppers exploit a variety of habitats, but lack the extreme specializations (“ecomorphs”) observed in other taxa (see Fig. 1). Accordingly, at a first glance, it seems that acridid grasshoppers exhibit a conservative and “all-purpose” morphology allowing them to perform equally well under different environments, which is contrary to one of the premises that are frequently used to catalogue a radiation as adaptive [19, 105]. However, in this study we provide evidence that natural selection could lead to a convergent body plan in very distant clades in this recent radiation [106]. Specifically, we found substrate type-related macroevolutionary variation in locomotory attributes and head morphology. The observed pattern was consistent with an evolutionary scenario with two adaptive peaks (selective agents) related to life-form (i.e., substrate type; plant vs. ground). Thus, these findings suggest that, although this radiation does not meet the conditions to be classified as adaptive sensu stricto (at least in comparison with textbook examples of rapid adaptive radiations such as Madagascan vangas and tetragnathid spiders on the Hawaiian Islands), it provides evidence that natural selection can act on a very small scale, and therefore it is not always detectable. So, even in a priori morphostatic radiations, adaptive processes can lead to subtle phenotypic variation. Hence, our results support the notion that ecological factors play a key role in evolutionary processes by acting as selective mechanisms and by imposing constraints that shape morphological traits [107]. In sum, this study highlights that natural selection, and not genetic drift, constitutes the main factor driving the disparification process in this recent radiation despite of the lack of apparent phenotypic and ecological variability. This calls into the question the use of term “non-adaptive” as synonymous with “morphostatic” since the total absence of an adaptive component seems to be unlikely in any radiation.

## Additional file

Additional file 1: Table S1. Information on substrate type (plant- or ground-perching) and niche breadth estimated in form of categorical (generalist vs. specialist species) and continuous (PDI: ‘Paired Difference Index’ values) variables for the 70 grasshopper taxa included in the present study. **Figure S1.** Relationship between relative femur length and relative femur width. Dots show mean values for each acridid species and colors indicate clade membership (blue: Gomphocerinae, red: Oedipodinae, yellow: Calliptaminae-Dericorythinae-Eyprepocnemidinae, grey: Catantopinae). The arrow denotes a case (*B. tryxalicerus*) that deviates remarkably (i.e., an outlier) from the general trend. **Figure S2.** Maximum likelihood ancestral reconstruction of head shape variation (PC*hs*) in Iberian short-horned grasshoppers. **Figure S3.** Maximum likelihood ancestral reconstruction of locomotory morphology (*p*PC*lm*) variation. **Figure S4.** Phylomorphospace projection of acridid species on the first two principal components of head shape variation, which account for 87% of the variance. For illustrative purposes, *B. tryxalicerus*, the most extreme case in both axes, was not represented. The inset shows the phylogenetic relationships among species including *B. tryxalicerus*, which is highlighted in color blue. **Figure S5.** Relationship between relative forewing length and femur width/length ratio. Dots show mean values for each acridid species and colors indicate clade membership (blue: Gomphocerinae, red: Oedipodinae, yellow: Calliptaminae-Dericorythinae-Eyprepocnemidinae, grey: Catantopinae). **Figure S6.** Head shape variation (PC*hs*) plotted against locomotory morphology variation (*p*PC*lm*)**.** Dots show mean values for each acridid species and colors indicate clade membership (blue: Gomphocerinae, red: Oedipodinae, yellow: Calliptaminae-Dericorythinae-Eyprepocnemidinae, grey: Catantopinae). **Figure S7.** The time-calibrated phylogeny for 70 species of short-horned grasshoppers used in the present study. (DOCX 517 kb)

## References

[1] Givnish TJ, Systma KJ (1997). Molecular evolution and adaptive radiation.

[2] Grant PR, Grant BR (2008). How and why species multiply: the radiation of Darwin’s finches.

[3] Simpson GG (1953). The major features of evolution.

[4] Schluter D (2000). The ecology of adaptive radiation.

[5] Losos JB (2010). Adaptive radiation, ecological opportunity, and evolutionary determinism. Am Nat.

[6] Harmon LJ, Losos JB, Davies J, Gillespie RG, Gittleman JL, Jennings WB, Kozak K, McPeek MA, Moreno-Roark F, Near TJ, Purvis A, Ricklefs RE, Schluter D, Schulte JA, Seehausen O, Sidlauskas B, Torres-Carvajal O, Weir JT, Mooers AØ (2010). Early bursts of body size and shape evolution are rare in comparative data. Evolution.

[7] Mahler DL, Revell LJ, Glor RE, Losos JB (2010). Ecological opportunity and the rate of morphological evolution in the diversification of greater Antillean anoles. Evolution.

[8] Rabosky DL, Lovette IJ (2008). Explosive evolutionary radiations: decreasing speciation or increasing extinction through time?. Evolution.

[9] Moen D, Morlon H (2014). Why does diversification slow down?. Trends Ecol Evol.

[10] Colombo M, Damerau M, Hanel R, Salzburger W, Matschiner M (2015). Diversity and disparity through time in the adaptive radiation of Antarctic notothenioid fishes. J Evol Biol.

[11] Price SA, Wainwright PC, Bellwood DR, Kazancioglu E, Collar DC, Near TJ (2010). Functional innovations and morphological diversification in parrotfish. Evolution.

[12] Rabosky DL, Santini F, Eastman J, Smith SA, Sidlauskas B, Chang J, Alfaro ME (2013). Rates of speciation and morphological evolution are correlated across the largest vertebrate radiation. Nat Commun.

[13] Claramunt S, Derryberry EP, Brumfield RT, Remsen JV (2012). Ecological opportunity and diversification in a continental radiation of birds: climbing adaptations and cladogenesis in the Furnariidae. Am Nat.

[14] Thacker CE (2014). Species and shape diversification are inversely correlated among gobies and cardinalfishes (Teleostei: Gobiiformes). Org Divers Evol.

[15] Derryberry EP, Claramunt S, Derryberry G, Chesser RT, Cracraft J, Aleixo A, Pérez-Emán J, Remsen JV, Brumfield RT (2011). Lineage diversification and morphological evolution in a large-scale continental radiation: the Neotropical ovenbirds and Woodcreepers (Aves: Furnariidae). Evolution.

[16] Hipsley CA, Miles DB, Müller J, Mu J (2014). Morphological disparity opposes latitudinal diversity gradient in lacertid lizards. Biol Lett.

[17] Alhajeri BH, Schenk JJ, Steppan SJ (2016). Ecomorphological diversification following continental colonization in muroid rodents (Rodentia: Muroidea). Biol J Linn Soc.

[18] Pigot AL, Phillimore AB, Owens I, Orme CDL (2010). The shape and temporal dynamics of phylogenetic trees arising from geographic speciation. Syst Biol.

[19] Glor RE (2010). Phylogenetic insights on adaptive radiation. Annu Rev Ecol Evol Syst.

[20] Simões M, Breitkreuz L, Alvarado M, Baca S, Cooper JC, Heins L, Herzog K, Lieberman BS (2016). The evolving theory of evolutionary radiations. Trends Ecol Evol.

[21] Seehausen O (2006). African cichlid fish: a model system in adaptive radiation research. Proc R Soc Lond B Biol Sci.

[22] Losos JB (2011). Convergence, adaptation, and constraint. Evolution.

[23] Frédérich B, Sorenson L, Santini F, Slater GJ, Alfaro ME (2013). Iterative ecological radiation and convergence during the evolutionary history of damselfishes (Pomacentridae). Am Nat.

[24] Pincheira-Donoso D, Harvey LP, Ruta M (2015). What defines an adaptive radiation? Macroevolutionary diversification dynamics of an exceptionally species-rich continental lizard radiation. BMC Evol Biol.

[25] Cameron RAD, Cook LM, Hallows JD (1996). Land snails on Porto Santo: adaptive and non-adaptive radiation. Philos Trans R Soc Lond Ser B Biol Sci.

[26] Wilke T, Benke M, Brändle M, Albrecht C, Bichain JM, Glaubrecht M (2010). The neglected side of the coin: non-adaptive radiations in spring snails (*Bythinella* spp.). Evolution in action.

[27] Wellenreuther M, Sánchez-Guillén RA (2015). Nonadaptive radiation in damselflies. Evol Appl.

[28] Gittenberger E (2004). Radiation and adaptation, evolutionary biology and semantics. Org Divers Evol.

[29] Mendelson TC, Shaw KL (2005). Sexual behaviour: rapid speciation in an arthropod. Nature.

[30] Olson ME, Arroyo-Santos A (2009). Thinking in continua: beyond the “adaptive radiation” metaphor. BioEssays.

[31] Gillespie R (2004). Community assembly through adaptive radiation in Hawaiian spiders. Science.

[32] Rowe KC, Aplin KP, Baverstock PR, Moritz C (2011). Recent and rapid speciation with limited morphological disparity in the genus *Rattus*. Syst Biol.

[33] Soulebeau A, Aubriot X, Gaudeul M, Rouhan G, Hennequin S, Haevermans T, Dubuisson JY, Jabbour F (2015). The hypothesis of adaptive radiation in evolutionary biology: hard facts about a hazy concept. Org Divers Evol.

[34] Song H, Amédégnato C, Cigliano MM, Desutter-Grandcolas L, Heads SW, Huang Y, Otte D, Whiting MF (2015). 300 million years of diversification: elucidating the patterns of orthopteran evolution based on comprehensive taxon and gene sampling. Cladistics.

[35] Mayer F, Berger D, Gottsberger B, Schulze W, Glaubrecht M (2010). Non-ecological radiations in acoustically communicating grasshoppers?. Evolution in action. Case studies in adaptive radiation, speciation and the origin of biodiversity.

[36] Kozak KH, Weisrock DW, Larson A (2006). Rapid lineage accumulation in a non-adaptive radiation: phylogenetic analysis of diversification rates in eastern north American woodland salamanders (Plethodontidae: Plethodon). Proc R Soc Lond B Biol Sci.

[37] Blankers T, Adams DC, Wiens JJ (2012). Ecological radiation with limited morphological diversification in salamanders. J Evol Biol.

[38] Moen DS, Irschick DJ, Wiens JJ (2013). Evolutionary conservatism and convergence both lead to striking similarity in ecology, morphology and performance across continents in frogs. Proc R Soc Lond B Biol Sci.

[39] Vidal-García M, Byrne PG, Roberts JD, Keogh JS (2014). The role of phylogeny and ecology in shaping morphology in 21 genera and 127 species of Australo-Papuan myobatrachid frogs. J Evol Biol.

[40] Vidal-García M, Keogh JS (2015). Convergent evolution across the Australian continent: ecotype diversification drives morphological convergence in two distantly related clades of Australian frogs. J Evol Biol.

[41] Edwards S, Tolley KA, Vanhooydonck B, Measey GJ, Herrel A (2013). Is dietary niche breadth linked to morphology and performance in Sandveld lizards Nucras (Sauria: Lacertidae)?. Biol J Linn Soc.

[42] Hewitt GM (1996). Some genetic consequences of ice ages, and their role in divergence and speciation. Biol J Linn Soc.

[43] Gómez A, Lunt DH, Weiss S, Ferrand N (2007). Refugia within refugia: patterns of phylogeographic concordance in the Iberian Peninsula. Phylogeography of southern European refugia.

[44] Presa JJ, García MD, Clemente ME (2007). Catalogue of Orthoptera Caelifera from the Iberian Peninsula and Balearic Islands (Orthoptera: Caelifera). J Orthoptera Res..

[45] Contreras D, Chapco W (2006). Molecular phylogenetic evidence for multiple dispersal events in gomphocerines grasshoppers. J Orthoptera Res..

[46] Nattier R, Robillard T, Amedegnato C, Couloux A, Cruaud C, Desutter-Grandcolas L (2011). Phylogeny and evolution of acoustic communication in Gomphocerinae (Orthoptera: Caelifera: Acrididae). Zool Scr.

[47] Katoh K, Standley DM (2013). MAFFT multiple sequence alignment software version 7: improvements in performance and usability. Mol Biol Evol.

[48] Vaidya G, Lohman DJ, Meier R (2011). SequenceMatrix: concatenation software for the fast assembly of multi-gene datasets with character set and codon information. Cladistics.

[49] Posada D (2008). jModelTest: phylogenetic model averaging. Mol Biol Evol.

[50] García-Navas V, Noguerales V. Cordero PJ, Ortego J. Ecological drivers of body size evolution and sexual size dimorphism in short-horned grasshoppers (Orthoptera: Acrididae). bioRxiv/2017/119560. doi:https://doi.org/10.1101/119560. 10.1111/jeb.13131 28609564

[51] Bazinet AL, Zwickl DJ, Cummings MP (2014). A gateway for phylogenetic analysis powered by grid computing featuring GARLI 2.0. Syst Biol.

[52] Drummond AJ, Suchard MA, Xie D, Rambaut A (2012). Bayesian phylogenetics with BEAUti and the BEAST 1.7. Mol Biol Evol.

[53] Fries M, Chapco W, Contreras D (2007). A molecular phylogenetic analysis of the Oedipodinae and their intercontinental relationships. J Orthoptera Res.

[54] Husemann M, Depperman J, Hochkirch A (2014). Multiple independent colonization of the Canary Islands by the winged grasshopper genus *Sphingonotus* Fieber, 1852. Mol Phylogenet Evol.

[55] Chapman RF, Joern A (1990). Biology of grasshoppers.

[56] Heidinger IMM, Hein S, Bonte D (2010). Patch connectivity and sand dynamics affect dispersal-related morphology of the blue-winged grasshopper *Oedipoda caerulescens* in coastal grey dunes. Insect Conserv Diver.

[57] Burrows M (2003). Froghopper insects leap to new heights. Nature.

[58] Burrows M, Sutton GP (2008). The effect of leg length on jumping performance of short- and long-legged leafhopper insects. J Exp Biol.

[59] Bennet-Clark HC, Chapman RF, Joern A (1990). Jumping in Orthoptera. Biology of grasshoppers.

[60] Revell LJ (2013). Two new graphical methods for mapping trait evolution on phylogenies. Methods Ecol Evol.

[61] Uyeda JC, Caetano DS, Pennell MW (2015). Comparative analysis of principal components can be misleading. Syst Biol.

[62] Uvarov BP (1977). Grasshoppers and locusts. A handbook of general Acridology vol 2.

[63] Yu Y, Zhang S, Zhang L, Zhao X (2009). Developmental expression of odorant-binding proteins and chemosensory proteins in the embryos of *Locusta migratoria*. Arch Insect Biochem Physiol.

[64] Chen HH, Zhao YX, Kang L (2003). Antennal sensilla of grasshoppers (Orthoptera: Acrididae) in relation to food preferences and habits. J Biosci.

[65] Barros FC, Herrel A, Kohldorf T (2011). Head shape evolution in Gymnophthalmidae: does habitat use constrain the evolution of cranial design in fossorial lizards?. J Evol Biol.

[66] Adams DC, Otarola-Castillo E (2013). *Geomorph*: an R package for the collection and analysis of geometric morphometric shape data. Methods Ecol Evol.

[67] Rohlf FJ, Slice DE (1990). Extensions of the Procrustes method for the optimal superimposition of landmarks. Syst Zool.

[68] Harz K (1975). Orthopteren Europas/the Orthoptera of Europe Vol. II.

[69] Llucià-Pomares D (2002). Revisión de los ortópteros (Insecta: Orthoptera) de Cataluña (España). Monografías SEA.

[70] Futuyma DJ, Moreno G (1998). The evolution of ecological specialization. Annu Rev Ecol Evol Syst.

[71] Devictor V, Clavel J, Julliard R, Lavergne S, Mouillot D, Thuiller W, Venail P, Villéger S, Mouquet N (2010). Defining and measuring ecological specialization. J Appl Ecol.

[72] Poisot T, Bever JD, Nemri A, Thrall PH, Hochberg ME (2011). A conceptual framework for the evolution of ecological specialisation. Ecol Lett.

[73] Poisot T, Thrall PH, Hochberg ME (2012). Trophic network structure emerges through antagonistic coevolution in temporally varying environments. Proc R Soc Lond B Biol Sci.

[74] Dormann CF, Fruend J, Gruber B. *Bipartite*: Visualising Bipartite Networks and Calculating Some (Ecological) Indices; 2016. https://cran.rproject.org/web/packages/bipartite/index.html.

[75] Pagel MD (1999). Inferring the historical patterns of biological evolution. Nature.

[76] Revell LJ (2011). *Phytools*: an R package for phylogenetic comparative biology (and other things). Methods Ecol Evol.

[77] Felsenstein J (1985). Phylogenies and the comparative method. Am Nat.

[78] Harmon LJ, Weir J, Brock C, Glor RE, Challenger W (2008). GEIGER: investigating evolutionary radiations. Bioinformatics.

[79] Orme D. CAPER: Comparative Analyses of Phylogenetics and Evolution in R. ver. 0.52; 2013. http://cran.r-project.org/web/packages/caper.

[80] Midford PE, Garland T Jr., Maddison WP. PDAP Package of Mesquite. Version 1.07; 2005. http://mesquiteproject.org/pdap_mesquite/.

[81] Sidlauskas B (2008). Continuous and arrested morphological diversification in sister clades of characiform fishes: a phylomorphospace approach. Evolution.

[82] Harmon LJ, Schulte JA, Larson A, Losos JB (2003). Tempo and mode of evolutionary radiation in iguanian lizards. Science.

[83] Freckleton RP, Harvey PH (2003). Detecting non-Brownian trait evolution in adaptive radiations. PLoS Biol.

[84] Beaulieu JM, Jhwueng D-C, Boettiger C, O’Meara BC (2012). Modeling stabilizing selection: expanding the Ornstein-Uhlenbeck model of adaptive evolution. Evolution.

[85] Butler MA, King AA (2004). Phylogenetic comparative analysis: a modelling approach for adaptive evolution. Am Nat.

[86] Berger D, Chobanov DP, Mayer F (2010). Interglacial refugia and range shifts of the alpine grasshopper *Stenobothrus cotticus* (Orthoptera: Acrididae: Gomphocerinae). Org Divers Evol.

[87] Gangwere SK, Morales-Agacino F (1990). The biogeography of Iberian orthopteroids. Miscelánea Zoológica..

[88] Bridle JR, Saldamando CI, Koning W, Butlin RK (2006). Assortative preferences and discrimination by females against hybrid male song in the grasshoppers Chorthippus brunneus and Chorthippus jacobsi (Orthoptera: Acrididae). J Evol Biol..

[89] Butlin RK, Beaumont M, Hewitt GM (1992). Selection for assortative mating between parapatric subspecies of grasshopper. Anim Behav..

[90] Hopkins MJ, Smith AB (2015). Dynamic evolutionary change in post-Paleozoic echinoids and the importance of scale when interpreting changes in rates of evolution. Proc Natl Acad Sci U S A.

[91] Ingram T, Harmon LJ, Shurin JB (2012). When should we expect early bursts of trait evolution in comparative data? Predictions from an evolutionary food web model. J Evol Biol.

[92] Wiens JJ, Ackerly DD, Allen AP, Anacker BL, Buckley LB, Cornell HV, Damschen EI, Davies TJ, Grytnes JA, Harrison SP, Hawkins BA, Holt RD, Mccain CM, Stephens PR (2010). Niche conservatism as an emerging principle in ecology and conservation biology. Ecol Lett.

[93] Münkemüller T, Boucher F, Thuiller W, Lavergne S (2015). Phylogenetic niche conservatism - common pitfalls and ways forward. Funct Ecol.

[94] Evans MEK, Smith SA, Flynn R, Donoghue MJ (2009). Climate, niche evolution, and diversification of the “bird-cage” evening primroses (*Oenothera*, sections *Anogra* and *Kleinia*). Am Nat.

[95] López-Fernández H, Arbour JH, Winemiller KO, Honeycutt RL (2013). Testing for ancient adaptive radiations in Neotropical cichlid fishes. Evolution.

[96] Toro E, Herrel A, Irschick D (2004). The evolution of jumping performance in Caribbean *Anolis* lizards: solutions to biomechanical trade-offs. Am Nat.

[97] Calsbeek R, Irschick DJ (2007). The quick and the dead: correlational selection on morphology, performance, and habitat use in island lizards. Evolution.

[98] Irschick DJ, Meyers JJ, Husak JF, Le Galliard JF (2008). How does selection operate on whole-organism functional performance capacities? A review and synthesis. Evol Ecol Res.

[99] Biewener A (2003). Animal Locomotion.

[100] Buckley TR, Attanayake D, Bradler S (2009). Extreme convergence in stick insect evolution: phylogenetic placement of the Lord Howe Island tree lobster. Proc R Soc Lond B Biol Sci.

[101] Gilman CA, Irschick DJ (2013). Foils of flexion: the effects of perch compliance on lizard locomotion and perch choice in the wild. Funct Ecol.

[102] Burrows M (2007). Anatomy of the hind legs and actions of their muscles during jumping in leafhopper insects. J Exp Biol.

[103] Endler JA (1978). A predator's view of animal color patterns. Evol Biol.

[104] Chapman RF (2003). Contact chemoreception in feeding by phytophagous insects. Annu Rev Entomol.

[105] Losos JB, Mahler DL, Bell MA, Futuyma DJ, Eanes WF, Levinon JS (2010). Adaptive radiation: the interaction of ecological opportunity, adaptation, and speciation. Evolution since Darwin: the first 150 years.

[106] Blom MPK, Horner P, Moritz C (2016). Convergence across a continent: adaptive diversification in a recent radiation of Australian lizards. Proc R Soc Lond B Biol Sci.

[107] Friedman ST, Price SA, Hoey AS, Wainwright PC (2016). Ecomorphological convergence in planktivorous surgeonfishes. J Evol Biol.

