# Supplementary material for: Phenotypic disparity in Iberian short-horned grasshoppers (Acrididae): the role of ecology and phylogeny
Source: BMC Evol Biol. 2017 May 4;17:109. doi: 10.1186/s12862-017-0954-7 (PMC5418863; doi:10.1186/s12862-017-0954-7)

**Additional file**

**Table S1.** Information on substrate type (plant- or ground-perching) and niche breadth estimated in form of categorical (generalist *vs*. specialist species) and continuous (PDI: ‘Paired Difference Index’ values) variables for the 70 grasshopper taxa included in the present study.

**Figure S1.** Relationship between relative femur length and relative femur width. Dots show mean values for each acridid species and colors indicate clade membership (blue: Gomphocerinae, red: Oedipodinae, yellow: Calliptaminae-Dericorythinae-Eyprepocnemidinae, grey: Catantopinae). The arrow denotes a case (*B. tryxalicerus*) that deviates remarkably (i.e., an outlier) from the general trend.

**Figure S2.** Maximum likelihood ancestral reconstruction of head shape variation (PC*hs*) in Iberian short-horned grasshoppers.

**Figure S3.** Maximum likelihood ancestral reconstruction of locomotory morphology (*p*PC*lm*) variation.

**Figure S4.** Phylomorphospace projection of acridid species on the first two principal components of head shape variation, which account for 87% of the variance. For illustrative purposes, *B. tryxalicerus*, the most extreme case in both axes, was not represented. The inset shows the phylogenetic relationships among species including *B. tryxalicerus*, which is highlighted in color blue.

**Figure S5.** Relationship between relative forewing length and femur width/length ratio. Dots show mean values for each acridid species and colors indicate clade membership (blue: Gomphocerinae, red: Oedipodinae, yellow: Calliptaminae-Dericorythinae-Eyprepocnemidinae, grey: Catantopinae).

**Figure S6.** Head shape variation (PC*hs*) plotted against locomotory morphology variation (*p*PC*lm*)**.** Dots show mean values for each acridid species and colors indicate clade membership (blue: Gomphocerinae, red: Oedipodinae, yellow: Calliptaminae-Dericorythinae-Eyprepocnemidinae, grey: Catantopinae).

**Figure S7.** The time-calibrated phylogeny for 70 species of short-horned grasshoppers used in the present study.

Table S1.

| **Taxa** | **Subfamily** | **Substrate type** | **Niche breadth** | **PDI** |
| --- | --- | --- | --- | --- |
| *Paracaloptenus bolivari* | Calliptaminae | ground | Generalist | 0.730 |
| *Calliptamus wattenwylianus* | Calliptaminae | ground | Generalist | 0.639 |
| *Calliptamus barbarus* | Calliptaminae | ground | Generalist | 0.391 |
| *Cophopodisma pyrenaea* | Catantopinae | ground | Specialist | 0.857 |
| *Podisma carpetana carpetana* | Catantopinae | ground | Generalist | 0.815 |
| *Podisma carpetana ignatii* | Catantopinae | ground | Generalist | 0.815 |
| *Podisma pedestris* | Catantopinae | ground | Generalist | 0.815 |
| *Pezotettix giornae* | Catantopinae | ground | Generalist | 0.631 |
| *Dericorys carthagonovae* | Dericorythinae | plant | Specialist | 1.000 |
| *Heteracris adspersa* | Eyprepocnemidinae | plant | Specialist | 1.000 |
| *Chorthippus apicalis* | Gomphocerinae | plant | Generalist | 0.642 |
| *Euchorthippus elegantulus gallicus* | Gomphocerinae | plant | Generalist | 1.000 |
| *Euchorthippus declivus* | Gomphocerinae | plant | Generalist | 0.677 |
| *Euchorthippus chopardi* | Gomphocerinae | plant | Generalist | 0.702 |
| *Euthystira brachyptera* | Gomphocerinae | plant | Specialist | 0.857 |
| *Pseudochorthippus montanus* | Gomphocerinae | plant | Generalist | 0.773 |
| *Chorthippus nevadensis* | Gomphocerinae | plant | Specialist | 1.000 |
| *Chorthippus* *binotatus binotatus* | Gomphocerinae | plant | Specialist | 0.785 |
| *Chorthippus saulcyi moralesi* | Gomphocerinae | plant | Specialist | 0.815 |
| *Chorthippus saulcyi saulcyi* | Gomphocerinae | plant | Specialist | 0.857 |
| *Chorthippus jacobsi* | Gomphocerinae | plant | Generalist | 0.487 |
| *Chorthippus vagans* | Gomphocerinae | plant | Generalist | 0.481 |
| *Chorthippus jucundus* | Gomphocerinae | plant | Specialist | 1.000 |
| *Stauroderus scalaris* | Gomphocerinae | plant | Generalist | 0.534 |
| *Gomphocerus sibiricus* | Gomphocerinae | plant | Specialist | 0.857 |
| *Pseudochorthippus parallelus erythropus* | Gomphocerinae | plant | Generalist | 0.630 |
| *Gomphoceridius brevipennis* | Gomphocerinae | ground | Generalist | 0.785 |
| *Stenobothrus lineatus* | Gomphocerinae | plant | Generalist | 0.773 |
| *Stenobothrus nigromaculatus* | Gomphocerinae | plant | Specialist | 0.815 |
| *Omocestus haemorrhoidalis* | Gomphocerinae | plant | Generalist | 0.730 |
| *Omocestus panteli* | Gomphocerinae | plant | Specialist | 0.887 |
| *Omocestus viridulus kaestneri* | Gomphocerinae | plant | Specialist | 0.815 |
| *Omocestus rufipes* | Gomphocerinae | plant | Generalist | 0.767 |
| *Omocestus viridulus viridulus* | Gomphocerinae | plant | Specialist | 0.815 |
| *Stenobothrus stigmaticus* | Gomphocerinae | ground | Specialist | 0.887 |
| *Omocestus femoralis* | Gomphocerinae | plant | Specialist | 0.857 |
| *Omocestus navasi* | Gomphocerinae | ground | Specialist | 0.887 |
| *Omocestus antigai* | Gomphocerinae | ground | Specialist | 0.887 |
| *Omocestus uhagonii* | Gomphocerinae | ground | Specialist | 0.928 |
| *Myrmeleotettix maculatus* | Gomphocerinae | ground | Generalist | 0.677 |
| *Omocestus minutissimus* | Gomphocerinae | ground | Specialist | 0.887 |
| *Omocestus bolivari* | Gomphocerinae | plant | Specialist | 0.857 |
| *Omocestus raymondi* | Gomphocerinae | ground | Generalist | 0.443 |
| *Stenobothrus bolivarii* | Gomphocerinae | plant | Specialist | 0.928 |
| *Stenobothrus fischeri* | Gomphocerinae | plant | Specialist | 0.857 |
| *Stenobothrus grammicus* | Gomphocerinae | ground | Specialist | 1.000 |
| *Stenobothrus festivus* | Gomphocerinae | plant | Generalist | 0.767 |
| *Dociostaurus crassiusculus* | Gomphocerinae | ground | Specialist | 0.815 |
| *Dociostaurus hispanicus* | Gomphocerinae | ground | Specialist | 0.928 |
| *Arcyptera fusca* | Gomphocerinae | ground | Generalist | 0.534 |
| *Dociostaurus jagoi occidentalis* | Gomphocerinae | ground | Generalist | 0.819 |
| *Dociostaurus maroccanus* | Gomphocerinae | ground | Specialist | 0.928 |
| *Ramburiella hispanica* | Gomphocerinae | plant | Specialist | 1.000 |
| *Brachycrotaphus tryxalicerus* | Gomphocerinae | plant | Specialist | 1.000 |
| *Stethophyma grossum* | Oedipodinae | ground | Specialist | 0.887 |
| *Calephorus compressicornis* | Oedipodinae | ground | Specialist | 1.000 |
| *Aiolopus strepens* | Oedipodinae | ground | Generalist | 0.338 |
| *Aiolopus puissanti* | Oedipodinae | ground | Specialist | 1.000 |
| *Paracinema tricolor bisignata* | Oedipodinae | plant | Specialist | 0.887 |
| *Sphingonotus azurescens* | Oedipodinae | ground | Generalist | 0.767 |
| *Sphingonotus imitans* | Oedipodinae | ground | Specialist | 1.000 |
| *Psophus stridulus* | Oedipodinae | ground | Specialist | 0.887 |
| *Mioscirtus wagneri maghrebi* | Oedipodinae | plant | Specialist | 1.000 |
| *Mioscirtus wagneri wagneri* | Oedipodinae | plant | Specialist | 1.000 |
| *Oedipoda fuscocincta caerulea* | Oedipodinae | ground | Generalist | 0.677 |
| *Oedipoda charpentieri* | Oedipodinae | ground | Generalist | 0.857 |
| *Oedipoda caerulescens* | Oedipodinae | ground | Generalist | 0.631 |
| *Morphacris fasciata* | Oedipodinae | ground | Specialist | 1.000 |
| *Oedaleus decorus* | Oedipodinae | ground | Generalist | 0.702 |
| *Acrotylus patruelis* | Oedipodinae | ground | Specialist | 1.000 |

Table S1 (continuation)

Figure S1


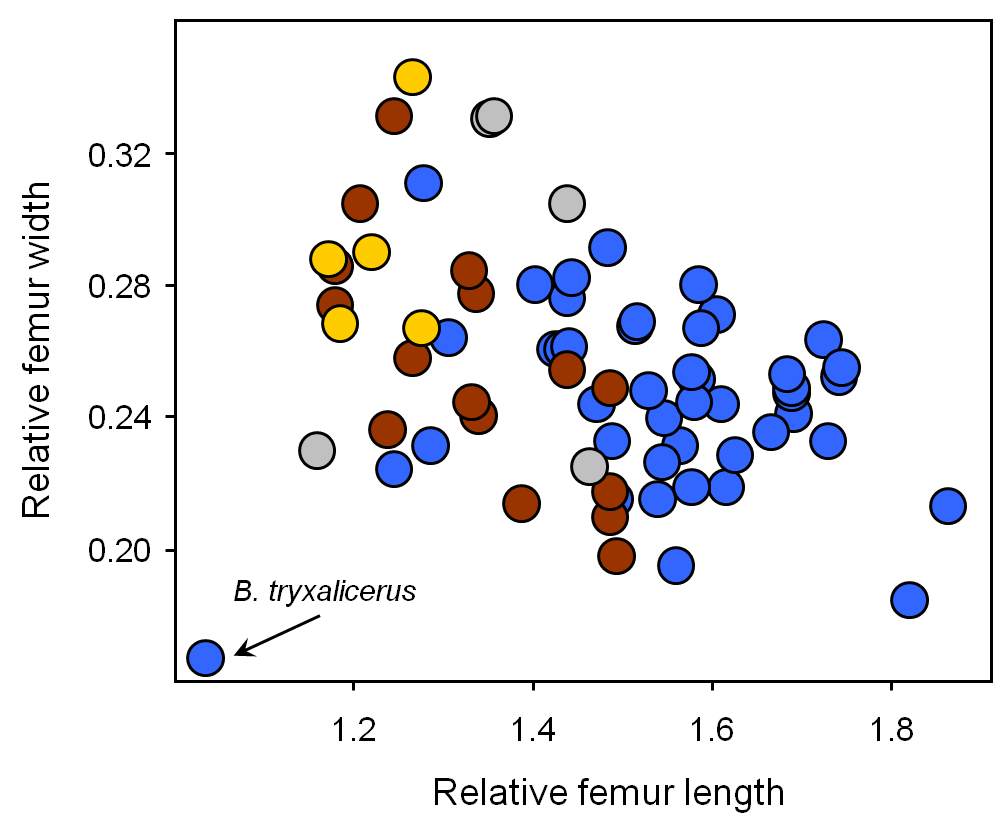


Figure S2


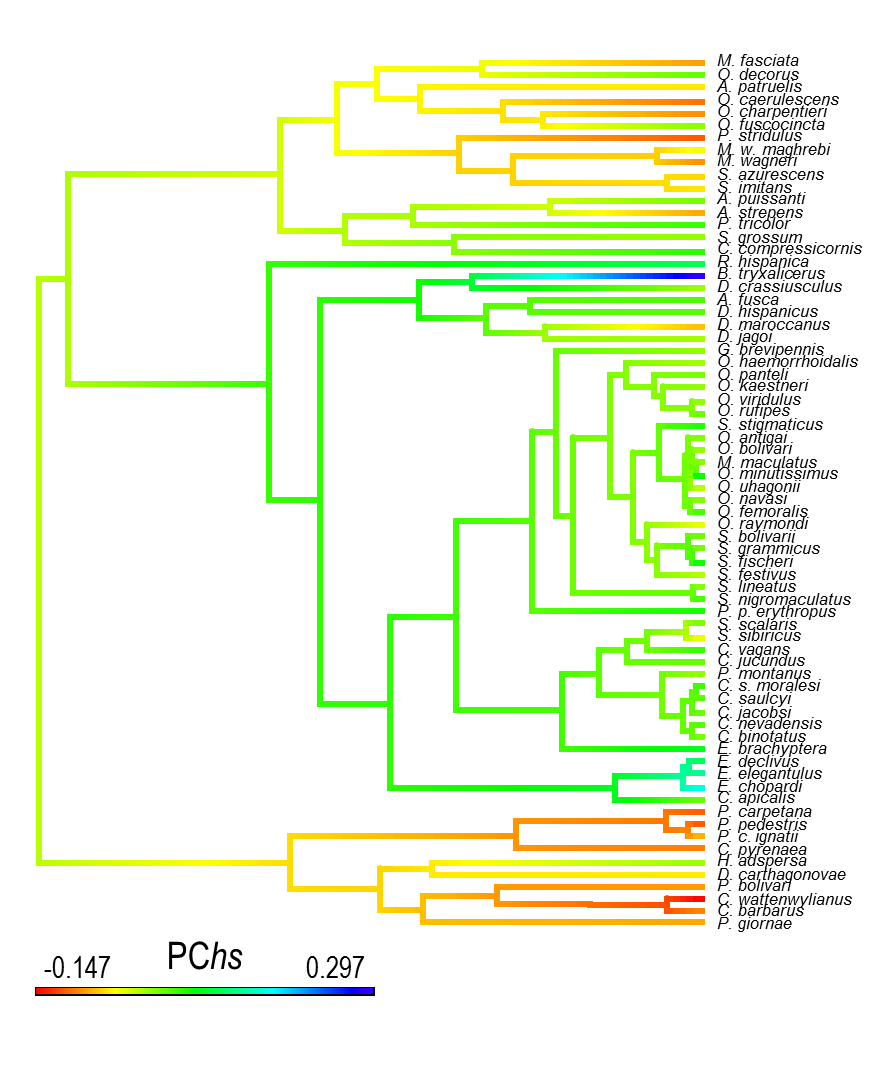


Figure S3


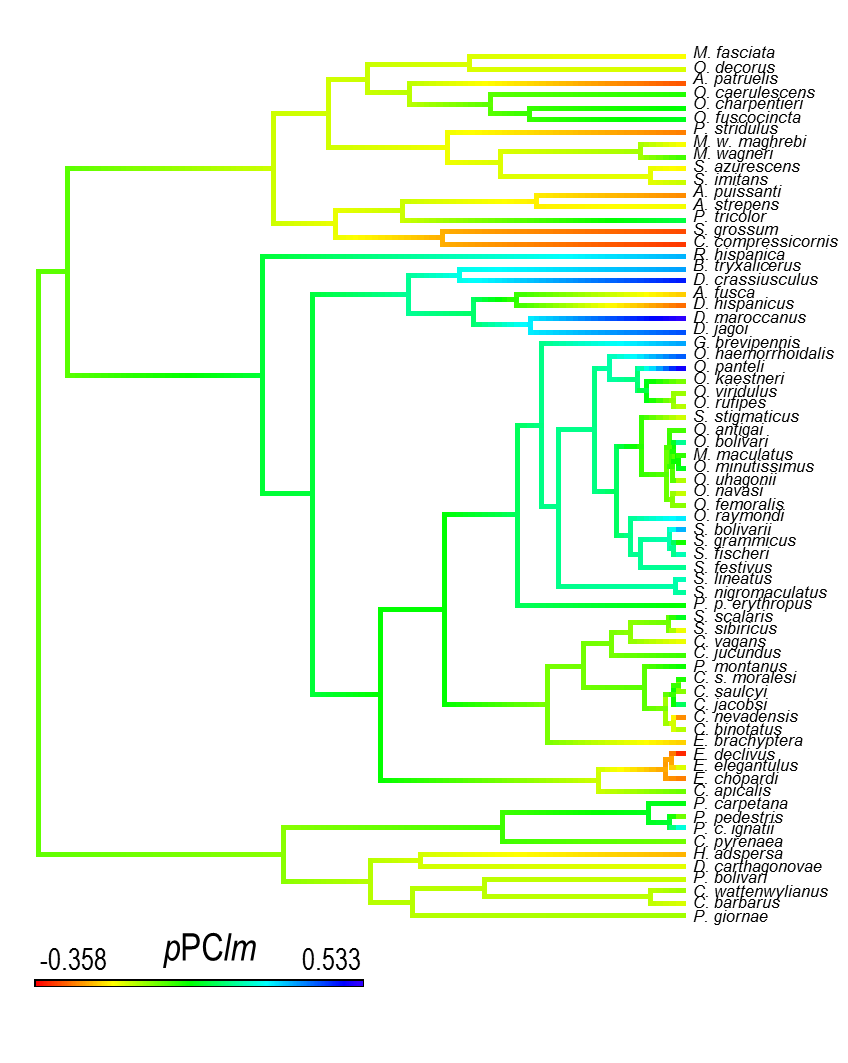


Figure S4


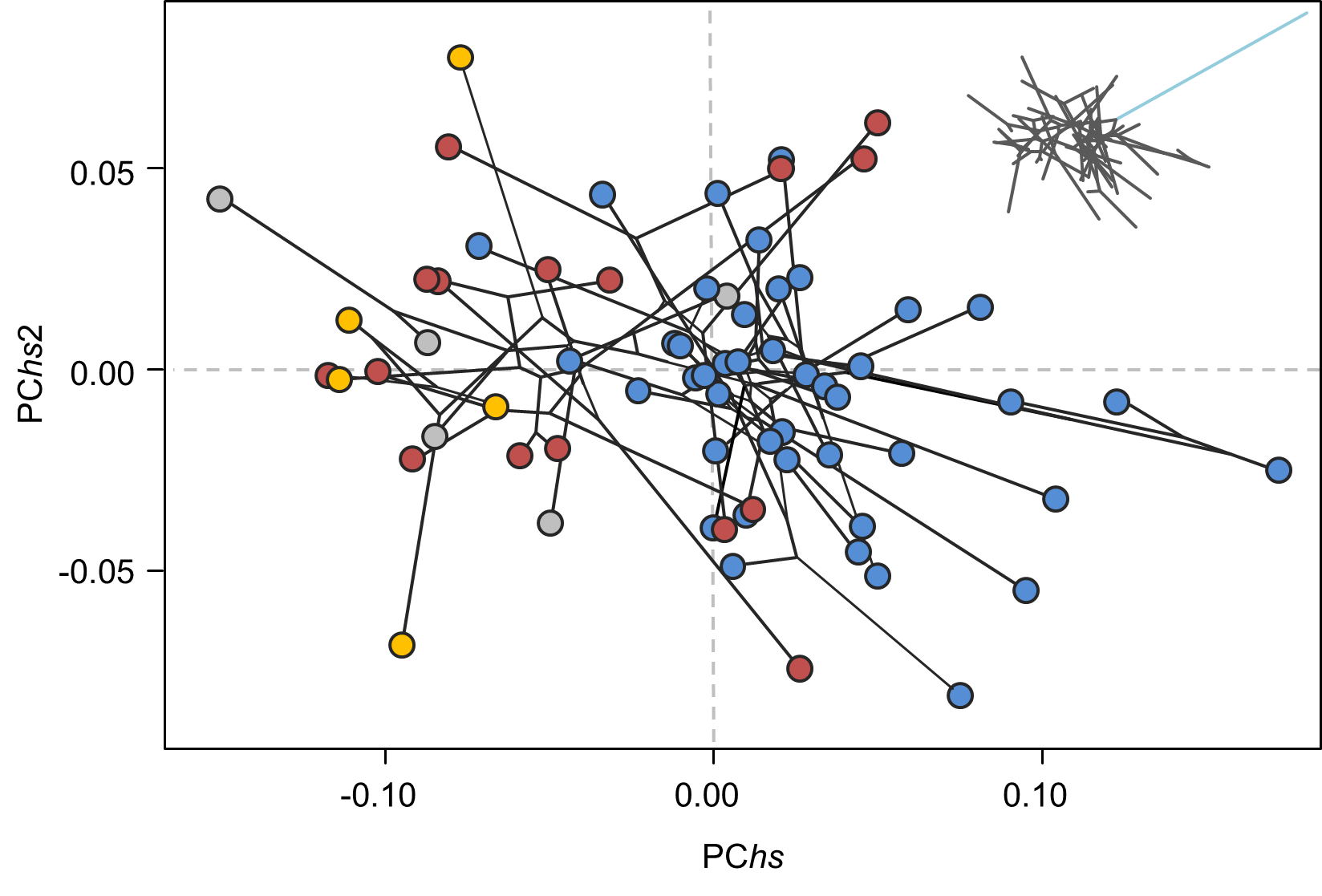
Figure S5


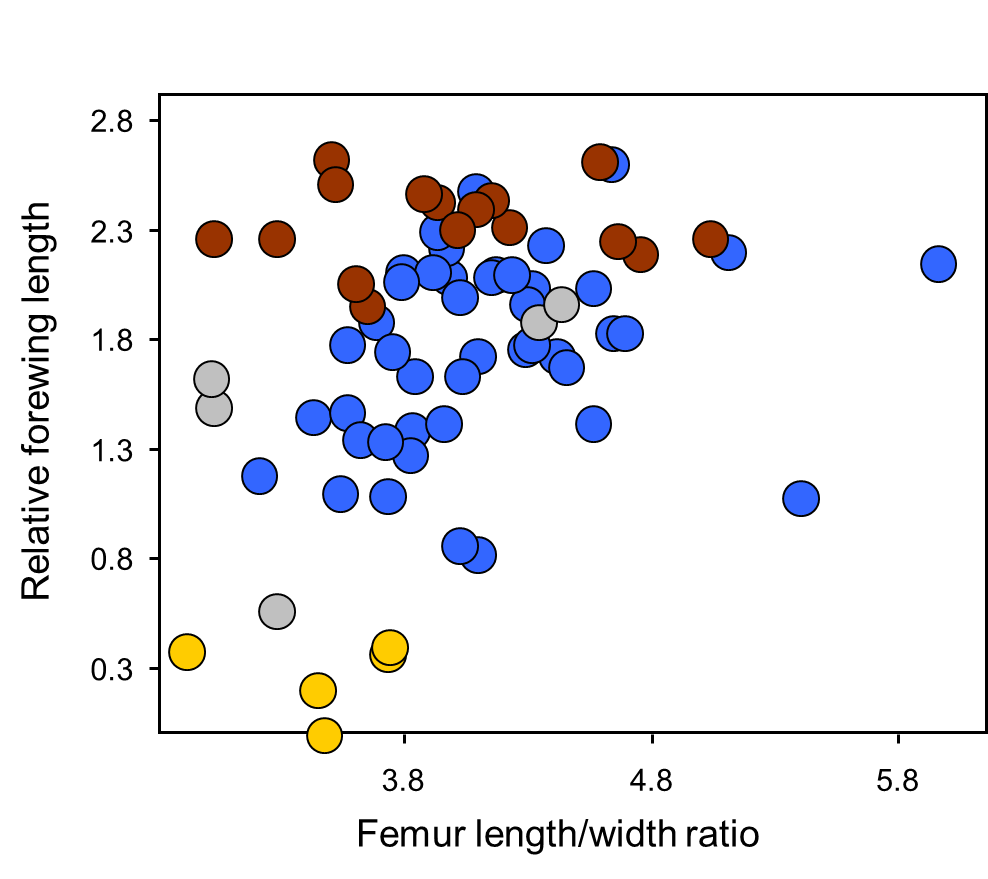


^
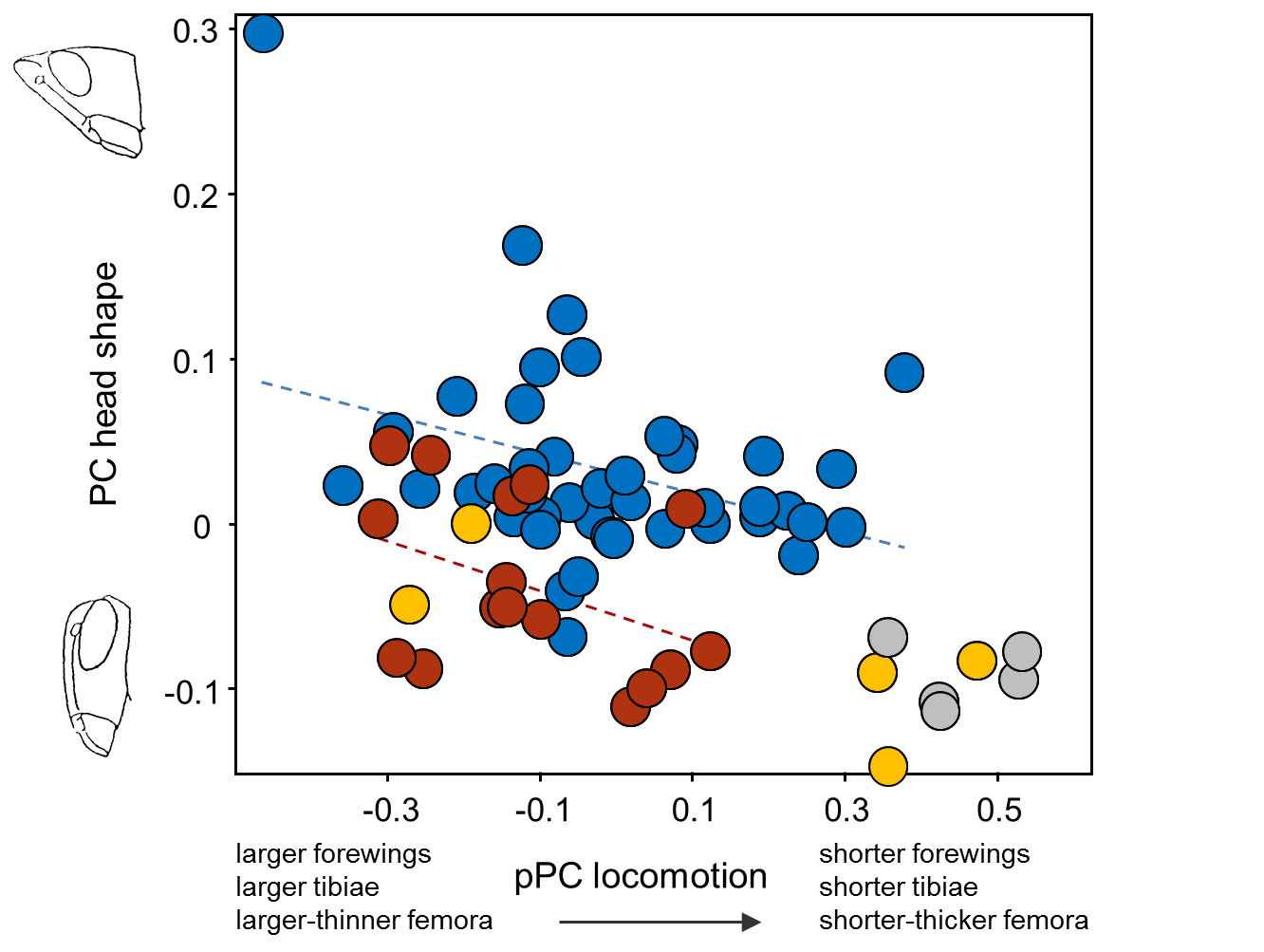
^Figure S6

Figure S7


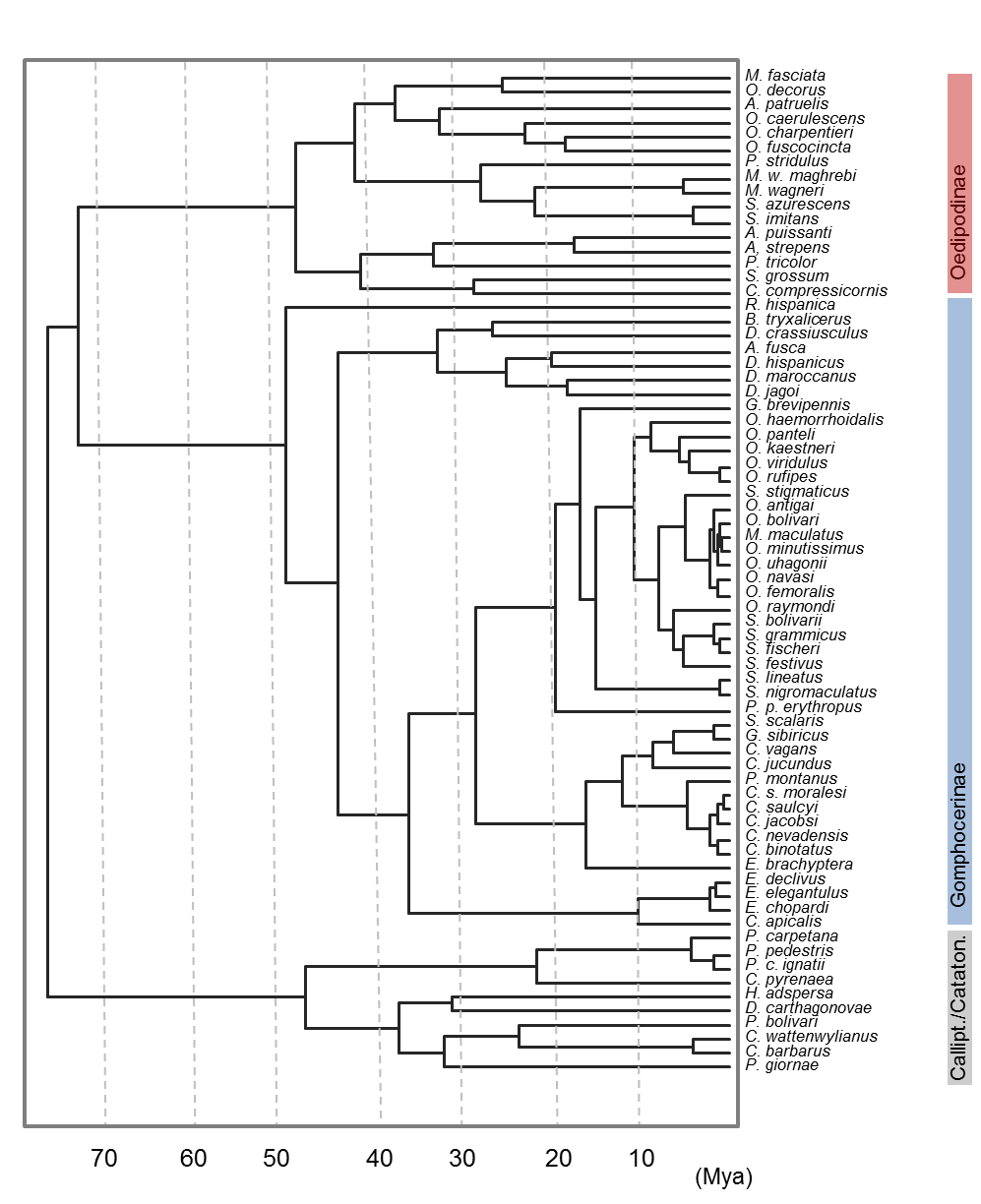

Supplement: Additional file 1: Table S1. — Information on substrate type (plant- or ground-perching) and niche breadth estimated in form of categorical (generalist vs. specialist species) and continuous (PDI: ‘Paired Difference Index’ values) variables for the 70 grasshopper taxa included in the present study. Figure S1. Relationship between relative femur length and relative femur width. Dots show mean values for each acridid species and colors indicate clade membership (blue: Gomphocerinae, red: Oedipodinae, yellow: Calliptaminae-Dericorythinae-Eyprepocnemidinae, grey: Catantopinae). The arrow denotes a case (B. tryxalicerus) that deviates remarkably (i.e., an outlier) from the general trend. Figure S2. Maximum likelihood ancestral reconstruction of head shape variation (PChs) in Iberian short-horned grasshoppers. Figure S3. Maximum likelihood ancestral reconstruction of locomotory morphology (pPClm) variation. Figure S4. Phylomorphospace projection of acridid species on the first two principal components of head shape variation, which account for 87% of the variance. For illustrative purposes, B. tryxalicerus, the most extreme case in both axes, was not represented. The inset shows the phylogenetic relationships among species including B. tryxalicerus, which is highlighted in color blue. Figure S5. Relationship between relative forewing length and femur width/length ratio. Dots show mean values for each acridid species and colors indicate clade membership (blue: Gomphocerinae, red: Oedipodinae, yellow: Calliptaminae-Dericorythinae-Eyprepocnemidinae, grey: Catantopinae). Figure S6. Head shape variation (PChs) plotted against locomotory morphology variation (pPClm). Dots show mean values for each acridid species and colors indicate clade membership (blue: Gomphocerinae, red: Oedipodinae, yellow: Calliptaminae-Dericorythinae-Eyprepocnemidinae, grey: Catantopinae). Figure S7. The time-calibrated phylogeny for 70 species of short-horned grasshoppers used in the present study. (DOCX 517 kb) [file 12862_2017_954_MOESM1_ESM.docx]
